# Natural and sexual selection drive multivariate phenotypic divergence along climatic gradients in an invasive fish

**DOI:** 10.1038/s41598-018-29254-4

**Published:** 2018-07-24

**Authors:** Xu Ouyang, Jiancao Gao, Meifeng Xie, Binghua Liu, Linjun Zhou, Bojian Chen, Jonas Jourdan, Rüdiger Riesch, Martin Plath

**Affiliations:** 1 College of Animal Science and Technology, Northwest A&F University, Yangling, Shaanxi 712100 P.R. China; 2 Department of River Ecology and Conservation, Senckenberg Research Institute and Natural History Museum Frankfurt, Gelnhausen, Germany; 3 School of Biological Sciences, Royal Holloway, University of London, Egham, Surrey, TW20 0EX UK

## Abstract

Invasive species that rapidly spread throughout novel distribution ranges are prime models to investigate climate-driven phenotypic diversification on a contemporary scale. Previous studies on adaptive diversification along latitudinal gradients in fish have mainly considered body size and reported either increased or decreased body size towards higher latitudes (i.e. Bergmann’s rule). Our study is the first to investigate phenotypic divergence in multiple traits, including sexually selected traits (size and shape of the male copulatory organ, the gonopodium) of invasive *Gambusia affinis* in China. We studied body size, life history traits and morphological variation across populations spanning 17 degrees of latitude and 16 degrees of longitude. Even though we found phenotypic variation along climatic gradients to be strongest in naturally selected traits, some sexually selected traits also showed systematic gradual divergence. For example, males from southern populations possessed wider gonopodia with increased armament. Generally, males and females diverged in response to different components of climatic gradients (latitudinal or longitudinal variation) and in different trait suites. We discuss that not only temperature regimes, but also indirect effects of increased resource and mate competition (as a function of different extrinsic overwinter mortality rates) alter the selective landscape along climatic gradients.

## Introduction

### Environmental variation and adaptation along climatic gradients

Identifying the ecological factors driving phenotypic diversification along climatic gradients lies at the heart of research in biogeography and evolutionary ecology^1–3^. The multivariate variation of ecological conditions along climatic gradients—especially in mean annual temperature^4^ and (daily or seasonal) temperature fluctuation^5,6^, but also in predation^7–10^ and other biotic factors^11,12^—creates divergent selective regimes that affect phenotypic traits directly related to fitness, including physiological, morphological, reproductive, and behavioral traits^13–16^. Studies over large geographic scales (in terms of longitudinal, latitudinal, and/or altitudinal variation) are likely to capture systematic variation not only in abiotic, but also in biotic selection factors and provide important insights into the mechanisms underlying the observed phenotypic divergence.

Adaptive phenotypic divergence along extensive climatic gradients has been reported for several taxa, including insects^17^, birds^18^ and mammals^19^. Phenotypic variation along latitudinal gradients received most scientific attention^20–22^. Variation in temperature regimes, precipitation, photo- and vegetation periods, to mention but some important abiotic factors, bring about an array of correlated changes in biotic selection factors (e.g., regarding species richness, primary production, and resource availability)^23,24^. Latitudinal variation in body size has been examined thoroughly^25–27^. Body size is linked to fitness as it not only influences physiological performance in contrasting thermal environments (with passive heat loss being reduced as the body volume-to-surface ratio increases^28^), but can also affect traits like anti-predator behavior (e.g., through altered maneuverability)^29–31^. In this context, Bergmann’s rule arguably represents the most widely known ecogeographic rule. It states that within a given taxonomic group of endotherms (populations, species, or higher taxonomic levels), larger body size would be predicted in colder environments, i.e., towards higher latitudes^32,33^. Bergmann’s rule has received extensive support from studies on different endotherms^34,35^, while the evidence for ectotherms is controversial^36–39^. For instance, revisiting *n* = 703 angling records from populations of 29 North American freshwater fishes, Rypel^40^ demonstrated that only 38% of species follow Bergmann’s rule, while 34% showed the reversed pattern, and the remaining 28% showed no intraspecific body size variation related to latitude. Another widely known ecogeographic law is Allen’s rule^41^, which states that the body extremities of endotherms that live under cold climatic conditions (i.e., at higher latitudes) are smaller than those of related taxa living at lower latitudes. Just like an increased body mass (i.e., Bergmann’s rule), shorter body appendages are thought to help increase the body volume-to-surface ratio, thereby minimizing thermal energy loss in cold environments^42–44^.

### Does sexual selection contribute to phenotypic divergence along climatic gradients?

Studies throughout the Animal Kingdom reported that body size is not only under natural but also sexual selection, e.g., via mate competition (intrasexual selection) or female mate choice (intersexual selection)^45–48^. More generally speaking, phenotypic traits typically considered to show latitudinal divergence in response to natural selection could also diverge—at least in part—through different forms of sexual selection. Regarding body size, this could be true especially for ectotherms, for which the above-mentioned form of natural selection from climatic variation does not readily apply^49^. Certain forms of sexual selection could be stronger at lower latitudes, where population densities and mate encounter rates can be higher^50,51^. However, the role played by sexual selection during phenotypic diversification along latitudinal gradients is generally not well understood.

Our present study provides novel insights into the potential contributions of both natural and sexual selection in driving phenotypic variation in invasive Western mosquitofish (*Gambusia affinis*) along climatic (latitudinal and longitudinal) gradients in the species’ invasive distribution range in China^52^. However, our study design was not suitable to tease apart the relative influences of natural and sexual selection on body size, and so investigating this question will be reserved to future studies. Still, we provide indirect evidence that both forms of selection are involved in phenotypic diversification along climatic gradients. Specifically, we demonstrate divergence in various phenotypic traits (male and female body size, body shape, and life histories), while including traits that are known to be under strong sexual selection.

### Multivariate phenotypic trait divergence in invasive mosquitofish

In an attempt to control mosquito-borne diseases, Western (*G. affinis*) and Eastern mosquitofish (*G. holbrooki*) have been introduced to at least 40 countries worldwide^53–55^, including the introduction of *G. affinis* to large parts of mainland China^52,56^. A recent study^57^ demonstrated that latitudinal body size variation of the closely related *G. holbrooki* in its native range in the Eastern USA is in support of Bergmann’s rule. Considering various ecological factors covarying with climate along the examined stretch of >14 degrees of latitude (such as the thermal regime, local population densities, and habitat productivity), a model selection approach identified the thermal regime as the main selection force driving the pattern of increasing body size with increasing latitude. Reproductive strategies showed *r*-selected life-history patterns at high latitudes [with high reproductive allocation (RA) and numerous small offspring], which could be owing to higher extrinsic mortality rates. On the other hand, other traits, like body condition and body shape, appear to diverge as a function of habitat productivity and population density. However, in another study^49^, *G. affinis* from 27 populations spanning nine degrees of latitude in North America showed a suggestive trend contradicting Bergmann’s rule. Finally, Stockwell and Vinyard^58^ studied life-history variation of four newly established (invasive) *G. affinis* populations and found small body size, early maturity, low fat reserves and small embryos in female *G. affinis* from thermally unstable environments.

**Figure 1:**
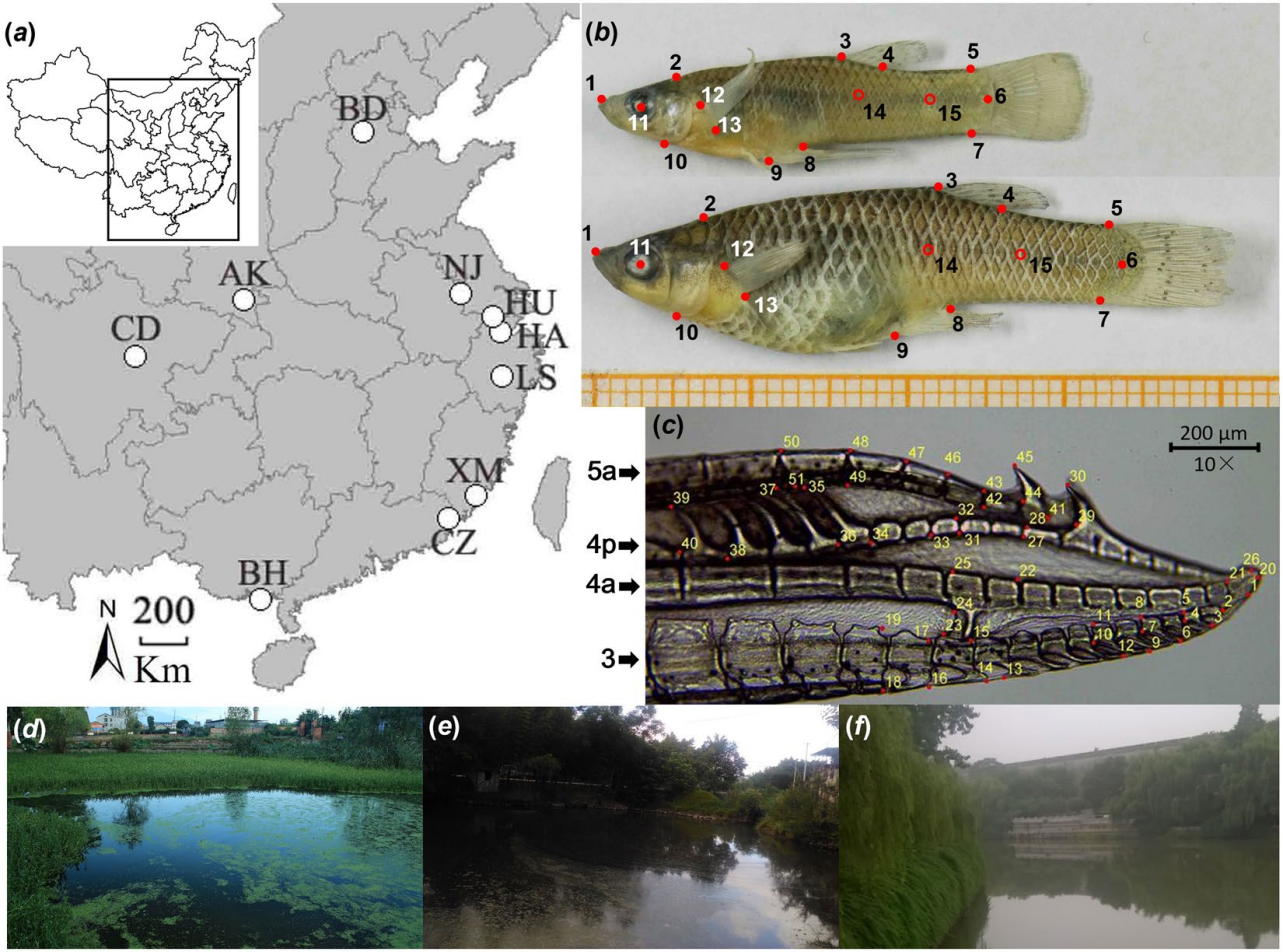
Sampling sites and morphological landmarks. (**a**) Ten sampling sites across China from which adult *G. affinis* were collected; city name codes can be found in Table 3. The map was generated using DAVI-GIS v 7.5.0 (http://www.diva-gis.org/). (**b**) Male (above) and female *G. affinis* (below) collected in Hangzhou in April 2016. Dots and numbers indicate the 13 landmarks used for morphometric analyses and two additional landmarks (14 and 15) used for the ‘unbending’ procedure in our Procrustes analyses. (**c**) Exemplary micrograph of a gonopodium showing the 51 morphometric landmarks. Nomenclature of fin rays (3, 4a, 4p, 5a) follows Rosen and Gordon^108^. (**d**–**f**) Exemplary photos of our sampling sites in Ankang, Chaozhou and Nanjing, respectively.

In this study, we collected invasive mosquitofish along 17 degrees of latitude and 16 degrees of longitude in mainland China (Fig. 1a). Based on existing theories and recent studies, we tested the following predictions:

1. Body size: Following a previous study in the congener *G. holbrooki*, we predicted that invasive *G. affinis* in China have larger body size at higher latitudes, partly because bigger individuals have an advantage in terms of greater overwintering survival in harsh environments^59,60^. On the other hand, body size could also show a pattern contradicting Bergmann’s rule^49^: life-history theory predicts that high adult mortality in fluctuating environments (i.e., higher latitudes, and continental/inland sites^61^) selects for early maturity and thus, small adult body size^62^. Moreover, lower resource availability in colder environments impairs growth rates^63^. We refrain from formulating predictions for body size evolution by sexual selection, but we will tentatively discuss our results in light of the insights into the general involvement of sexual selection in driving trait divergence, as obtained from our analyses of the size and shape of the distal part of the male intromittant organ, called gonopodium^64,65^.
2. Life-history traits: *G. affinis* at higher latitudes are likely to experience high overwinter mortality^66,67^. Other environmental factors, such as fluctuating productivity^68^, should increase (unpredictable) mortality rates. Based on life-history theory^62,69^, we predicted *G. affinis* females to produce more but smaller offspring at higher latitudes, and to have a higher total investment into reproduction. More stable and benign conditions at lower latitudes likely result in higher survival and continuously higher population densities. Increased intraspecific competition should favor the production of fewer but bigger offspring, which are more competitive^62,70^.
3. Morphology: Since female body shape is tightly linked to life-history traits^71–73^, we predicted that divergence in female body shape largely follows patterns predicted for life-history divergence. Populations at higher latitudes—characterized by higher reproductive effort—should have enlarged abdomens to harbor larger broods, more anteriorly positioned pectoral fins and relatively smaller heads than more southern populations. By contrast, males are unlikely to show a similar degree of morphological divergence mirroring life-history divergence.
4. Gonopodium morphology: Populations from lower latitudes and from more coastal areas (which are preferred by mosquitofish^53^) likely experience more stable and benign environmental conditions. Low overwinter mortality should result in higher population densities and heightened intrasexual competition amongst males. Also, mosquitofish females prefer males with longer gonopodia^64^, and females are more likely to exert mate choice when population densities are high, as they have more opportunities to choose (even though this effect may be weakened by coercive mating^74–77^). This could result in elongated gonopodia (via female choice) and more rigid distal gonopodium tips (a trait that is beneficial to achieve coercive copulations^65^) towards lower latitudes and in coastal areas.

## Results

### Population genetic analyses

**Figure 2:**
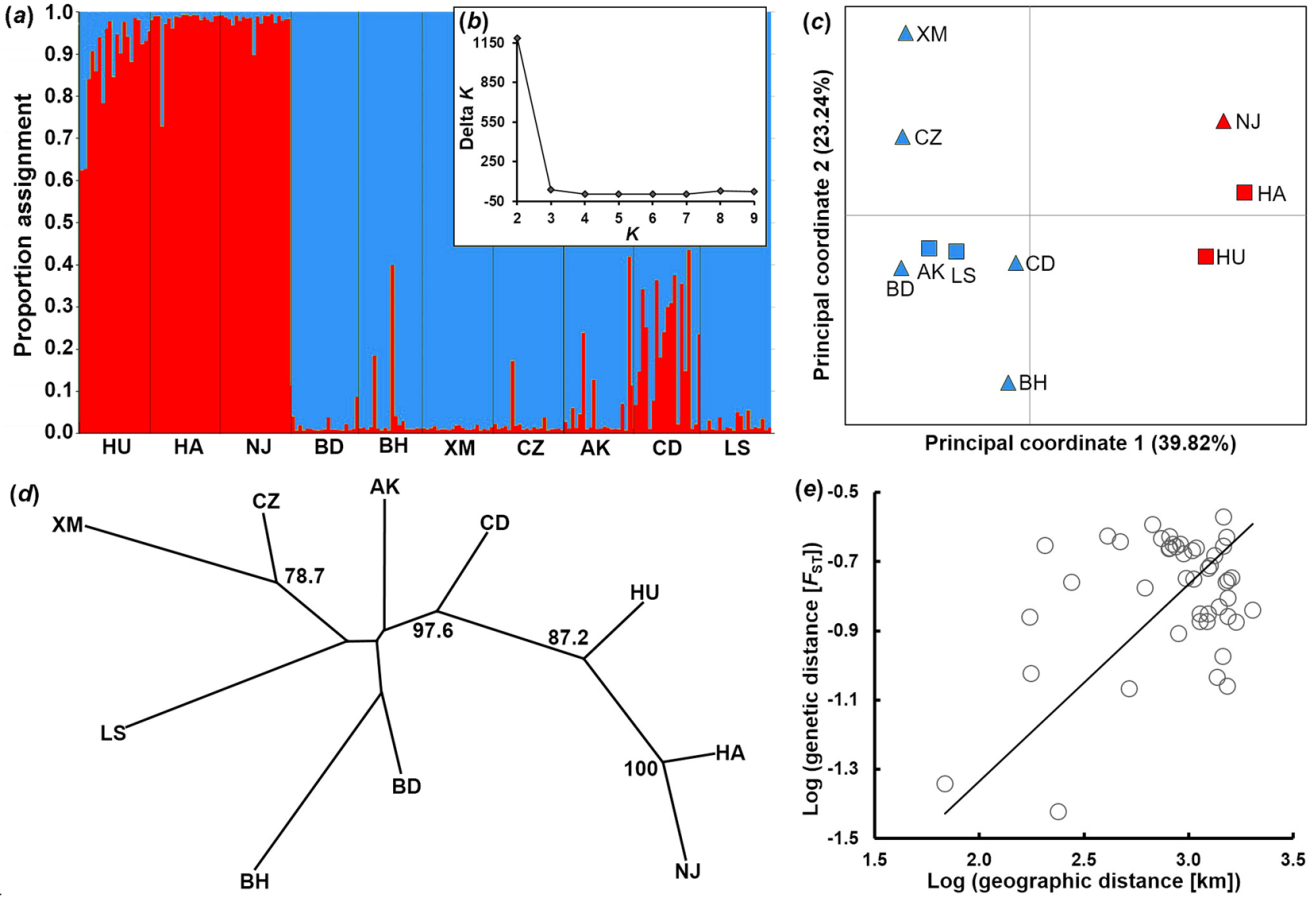
Population genetic analyses of invasive *G. affinis* in mainland China. (**a**) Genetic structure among populations (see Table 1 for population codes). Individual assignment to two genetically distinct clusters using STRUCTURE^168^. Likelihood of assignment for each individual is shown as a vertical bar. (**b**) Bayesian inference of the number of genetically distinct clusters (*K*) among the 10 sampled populations using Δ*K*^169^. (**c**) Principal coordinate analysis (PCoA) showing genetic differentiation between populations according to the first two axes. Percent variance explained is given in parentheses. Triangles indicate positive values of the third axis (12.22% variance explained), while squares indicate negative values. Colors signify assignment to *K* = 2 genetically distinct clusters in STRUCTURE. (**d**) Neighbor-joining tree based on genetic distances (Nei’s *D*_A_). Numbers at nodes indicate bootstrap support; only values >70 are presented. (**e**) Correlation between genetic distance and geographic distance. A Mantel test on log-transformed pairwise genetic distances (*F*_ST_-values) detected a significant effect of log-transformed geographic distances (Z = −103.73, *r* = 0.37, one-sided *P* = 0.014).

We conducted population genetic analyses based on 15 nuclear microsatellites^78–80^. This part of our study served not only as a validation of species identity^56^, but also tested for ‘unusual’ patterns of population genetic structure (suggesting recent translocations or multiple introductions)—important background information for the interpretation of our data on phenotypic divergence. Standard population genetic parameters for each population can be found in Table S1. We found varying degrees of genetic differentiation between populations, ranging from virtual panmixis (*F*_ST_ = 0.038, between Nanjing and Hangzhou) to moderate genetic differentiation (*F*_ST_ = 0.268, between Xiamen and Beihai). We detected *K* = 2 to be the most likely number of genetically distinct clades in our STRUCTURE analysis (Fig. 2a,b). This result was also reflected by our principal coordinate analysis, in which the same three populations (Nanjing, Hangzhou and Huzhou) formed a distinct cluster (Fig. 2c). While this pattern could indicate presence of two species of *Gambusia* in China (i.e., *G. affinis* and *G. holbrooki*), a neighbor-joining tree based on Nei’s *D*_A_ demonstrated that genetic distances between population pairs (mean ± SD = 0.265 ± 0.082) were in the range of within-species variability (Fig. 2d; see also^63^). Our results are congruent with previous studies suggesting that invasive *G. affinis* may have been introduced to China through two possible routes^56,81–84^. Most importantly, a Mantel test revealed that genetic distances were significantly correlated with geographic distances (*Z* = −103.73, *r* = 0.37, one-sided *P* = 0.014; Fig. 2e), suggesting either gradual translocation in a stepping-stone-like fashion, or at least some degree of ongoing gene-flow between populations.

According to bottleneck analyses under three microsatellite evolution models, most of the populations underwent genetic bottlenecks in the recent past—especially the populations from Ankang, Xiamen and Nanjing (Table S2), which implies that genetic drift could have played an additional role in creating phenotypic divergence among study sites.

### Phenotypic divergence

We used principle component analysis (PCA) to condense various climatic data into two climate-related principle components (PCs). PC1 described the gradient from northern towards southern sites (latitudinal variation), while PC2 described gradual changes from coastal towards inland sites (longitudinal variation; Table 1). The two climate-related PCs were used as covariates in subsequent analyses to test for phenotypic divergence along climatic gradients.

**Table 1:** Results of a correlation matrix-based principle component analysis (PCA).

	Principal components	
	1	2
Eigenvalue	4.02	1.97
Variance explained [%]	57.42	28.12
Mean annual temperature	**0.840**	−0.404
Max. temperature of the warmest month	**−0.810**	−0.251
Min. temperature of the coldest month	**0.934**	−0.138
Annual temperature difference	**−0.943**	0.025
Annual precipitation	**0.730**	−0.562
Altitude	−0.052	**0.891**
Distance to the sea	−0.036	**0.987**

Input variables were climatic and geographic variables. Shown are axis loadings for PCs with eigenvalues ≥1.0; axis loadings ≥|0.6| are highlighted in bold font.

**Figure 3:**
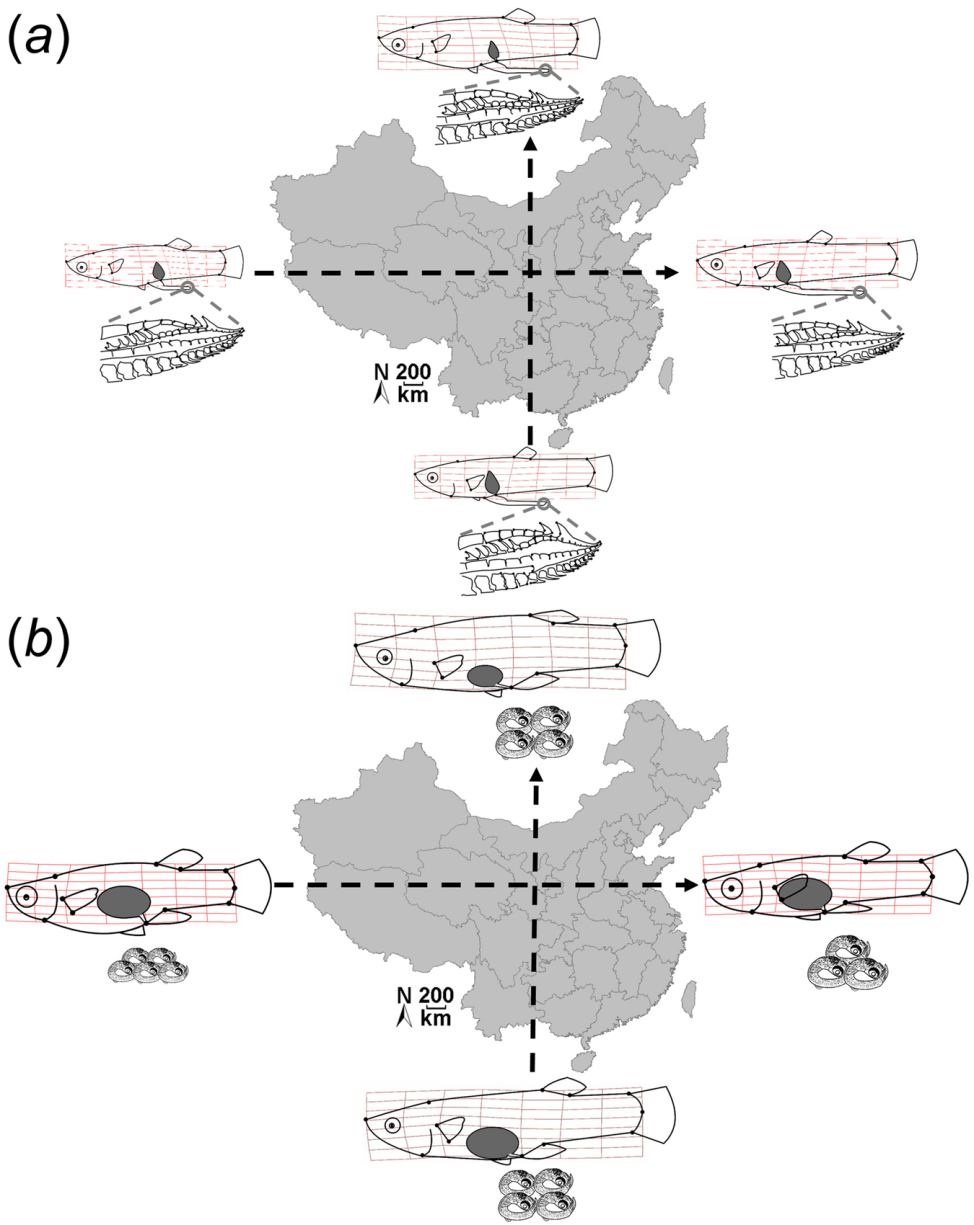
Phenotypic diversification in (**a**) male and (**b)** female *G. affinis* along climatic gradients. Visualization of the main results from *post-hoc* ANCOVAs on single traits (see main text). This figure serves as a graphic overview and summary of the effects that are presented in detail in subsequent sections of the results and are depicted in Figs 4–10. Illustrated are differences in overall body size, body shape, reproductive vs. somatic investment, the offspring size/fecundity trade-off, and gonopodium tip morphology along climate-related PC1 (from south to north) and PC2 (from inland to coastal sites).

We used analyses of covariance (ANCOVAs; for male and female SL, and gonopodium length) and multivariate analyses of covariance (MANCOVAs; for all other trait suites) to test if the different phenotypic character suites under investigation show gradual variation along both climatic gradients (PC1 and PC2) and found significant effects of at least one covariate in all models but the ANCOVA on female body size (Table 2b). Overall, the analysis of male body size yielded weak effects based on partial eta squared (*η*_p_^2^) of significant model terms (0.064–0.140). Strong effects were seen in the case of life-history traits (males: 0.210–0.327; females: 0.287–0.332). Body shape showed the strongest effects when comparing all models (males: 0.320–0.363; females: 0.311–0.384). Considerably weaker effects were found for variation in gonopodium morphology (0.120–0.164), and the analysis of gonopodium length yielded the weakest effect (0.051; Table 2). A visualization of our main results from subsequent analyses of single traits (i.e. *post-hoc* ANCOVAs using the same model structure as in the main MANCOVAs) is provided in Fig. 3. The results of alternative analytical models including ‘population’ as a fixed factor instead of climate-related PCs are shown in Supplementary Table S3.

**Table 2:** Results from (multivariate) analyses of covariance (M)ANCOVA.

	Source	d.f.	*F*	*P*	Partial *η*^2^	Variance explained [%]
(*a*) Male standard length	**Climatic PC1**	**1**	**12.339**	<**0.001**	**0.064**	**45.71**
	**Climatic PC2**	**1**	**29.439**	<**0.001**	**0.140**	**100.00**
	(Climatic PC1 × PC2)	1	1.084	0.299	0.006	—
	Error	180	—	—	—	—
(*b*) Female standard length	Climatic PC1	1	3.61	0.059	0.019	100.00
	Climatic PC2	1	0.129	0.720	0.001	5.26
	(Climatic PC1 × PC2)	1	1.824	0.178	0.010	—
	Error	187	—	—	—	—
(*c*) Male life-history traits	**SL**	**3**	**621.789**	<**0.001**	**0.913**	**100.00**
	**Climatic PC1**	**3**	**24.126**	<**0.001**	**0.290**	**31.76**
	**Climatic PC2**	**3**	**15.643**	<**0.001**	**0.210**	**23.00**
	**Climatic PC1** × **PC2**	**3**	**28.611**	<**0.001**	**0.327**	**35.82**
	Error	177	—	—	—	—
(*d*) Female life-history traits	**SL**	**6**	**389.525**	<**0.001**	**0.928**	**100.00**
	Stage of development	6	0.883	0.509	0.029	6.40
	**Climatic PC1**	**6**	**14.843**	<**0.001**	**0.331**	**20.39**
	**Climatic PC2**	**6**	**12.103**	<**0.001**	**0.287**	**38.29**
	**Climatic PC1** × **PC2**	**6**	**14.891**	<**0.001**	**0.332**	**16.27**
	Error	180	—	—	—	—
(*e*) Male morphology-related PCs	**Centroid size**	**10**	**9.356**	<**0.001**	**0.346**	**95.31**
	**Climatic PC1**	**10**	**10.002**	<**0.001**	**0.361**	**99.45**
	**Climatic PC2**	**10**	**8.327**	<**0.001**	**0.320**	**88.15**
	**Climatic PC1** × **PC2**	**10**	**10.093**	<**0.001**	**0.363**	**100.00**
	Error	177	—	—	—	—
(*f*) Female morphology-related PCs	**Centroid size**	**10**	**2.575**	**0.006**	**0.116**	**30.21**
	**Climatic PC1**	**10**	**8.892**	<**0.001**	**0.311**	**80.99**
	**Climatic PC2**	**10**	**12.269**	<**0.001**	**0.384**	**100.00**
	**Climatic PC1** × **PC2**	**10**	**9.700**	<**0.001**	**0.330**	**85.94**
	Error	197	—	—	—	—
(*g*) Gonopodium morphology-related PCs	**Centroid size**	**9**	**18.910**	<**0.001**	**0.502**	**100.00**
	**Gonopodium length**	**9**	**8.919**	<**0.001**	**0.322**	**64.14**
	**Climatic PC1**	**9**	**2.880**	**0.003**	**0.133**	**26.49**
	**Climatic PC2**	**9**	**3.672**	<**0.001**	**0.164**	**32.67**
	**Climatic PC1** × **PC2**	**9**	**2.565**	**0.009**	**0.120**	**23.90**
	Error	169	—	—	—	—
(*h*) Gonopodium length	**SL**	**1**	**537.627**	<**0.001**	**0.735**	**100.00**
	Climatic PC1	1	0.236	0.627	0.001	0.14
	**Climatic PC2**	**1**	**14.654**	<**0.001**	**0.070**	**9.52**
	(Climatic PC1 × PC2)	1	0.493	0.483	0.003	0.41
	Error	179	—	—	—	—

ANCOVAs used (*a*) male and (*b*) female standard length as dependent variables, while MANCOVAs used (*c*) male and (*d*) female life-history traits, (*e*) male and (*f*) female morphology-related PCs, and (*g*) gonopodium morphology-related PCs as dependent variables. (*h*) ANCOVA on gonopodium length. Climatic information was included in the form of two covariates (‘climatic PCs’, see Table 2). We included ‘standard length’ (SL), ‘centroid size’, and ‘stage of development’ as additional covariates where applicable. Statistically significant effects are shown in bold font. Interaction terms in parentheses indicate that the term was removed from the final model since it had no significant effect in the full model. Relative variance explained was calculated from Wilk’s partial *η*^2^.

#### Body size

To assess divergence of body size along climatic gradients, we ran ANCOVAs for each sex using the two climate-related PCs (see above) as covariates. We found climate-related PC2 (*η*_p_^2^ = 0.140) and PC1 (*η*_p_^2^ = 0.064) to have significant effects on male body size (Table 2a), while females showed no significant variation along both climatic gradients (*P* > 0.059; Table 2b).

**Figure 4:**
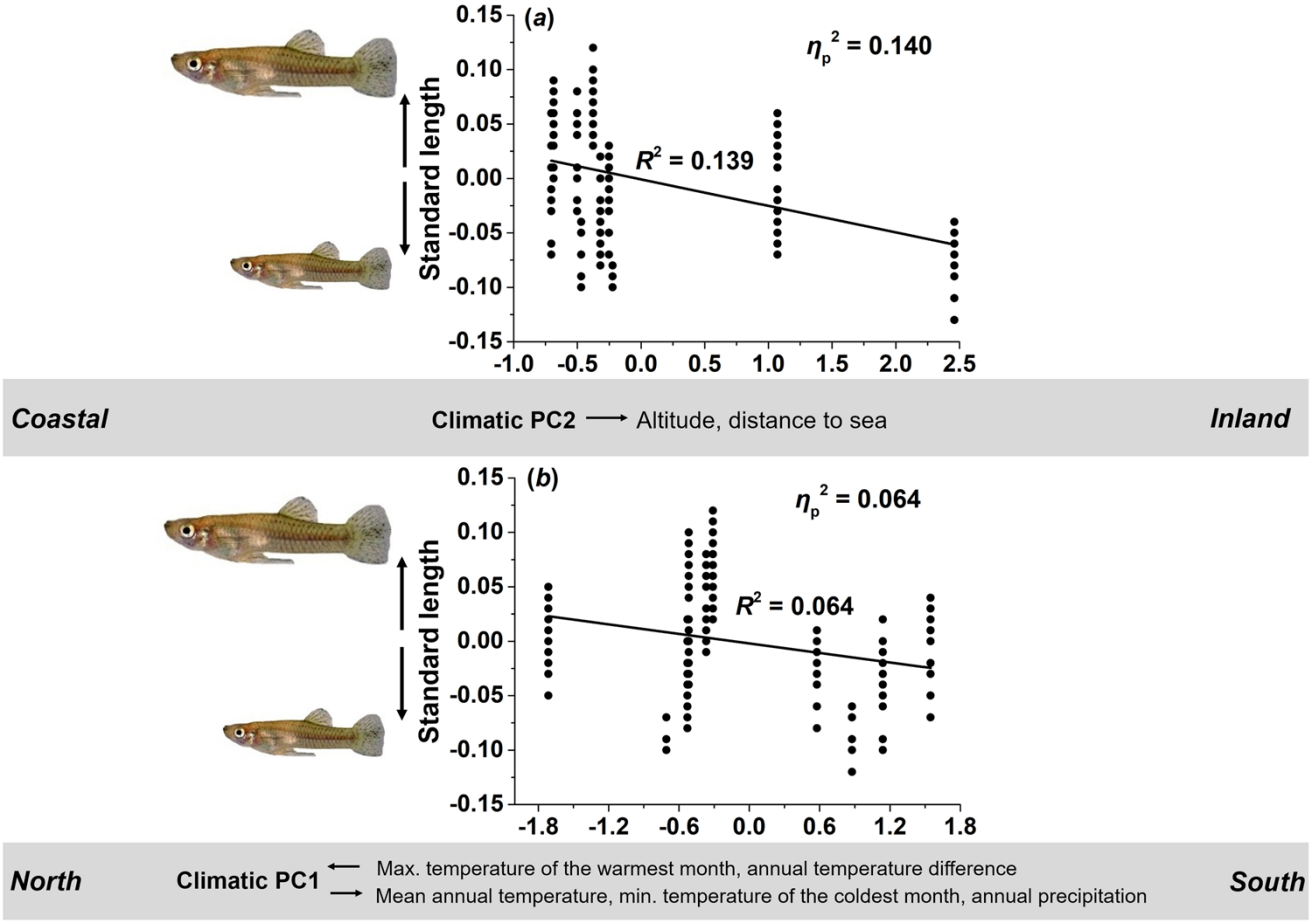
Scatter plot and linear fit of male body size along climatic gradients. Visualization of the main effect of both climatic PCs on male standard length (residuals, corrected for the other model term; see Table 2a). Males showed decreased body size along (**a**) climatic PC2 and (**b**) PC1.

**Climate-related PC2:** The effect of PC2 on male body size reflects that males became smaller from coastal towards inland sites (*R*^2^ = 0.139; Fig. 4a).

**Climate-related PC1:** The effect of PC1 suggests that males from more southern populations were smaller than populations from northern sampling sites (*R*^2^ = 0.064; Fig. 4b).

#### Life-history variation

We used sex-specific MANCOVAs to examine variation in male and female life-history traits along both climatic gradients (PC1 and PC2). Subsequently, we ran separate *post-hoc* ANCOVAs on each life-history trait to identify which traits contributed to significant model terms.

### Males

**Figure 5:**
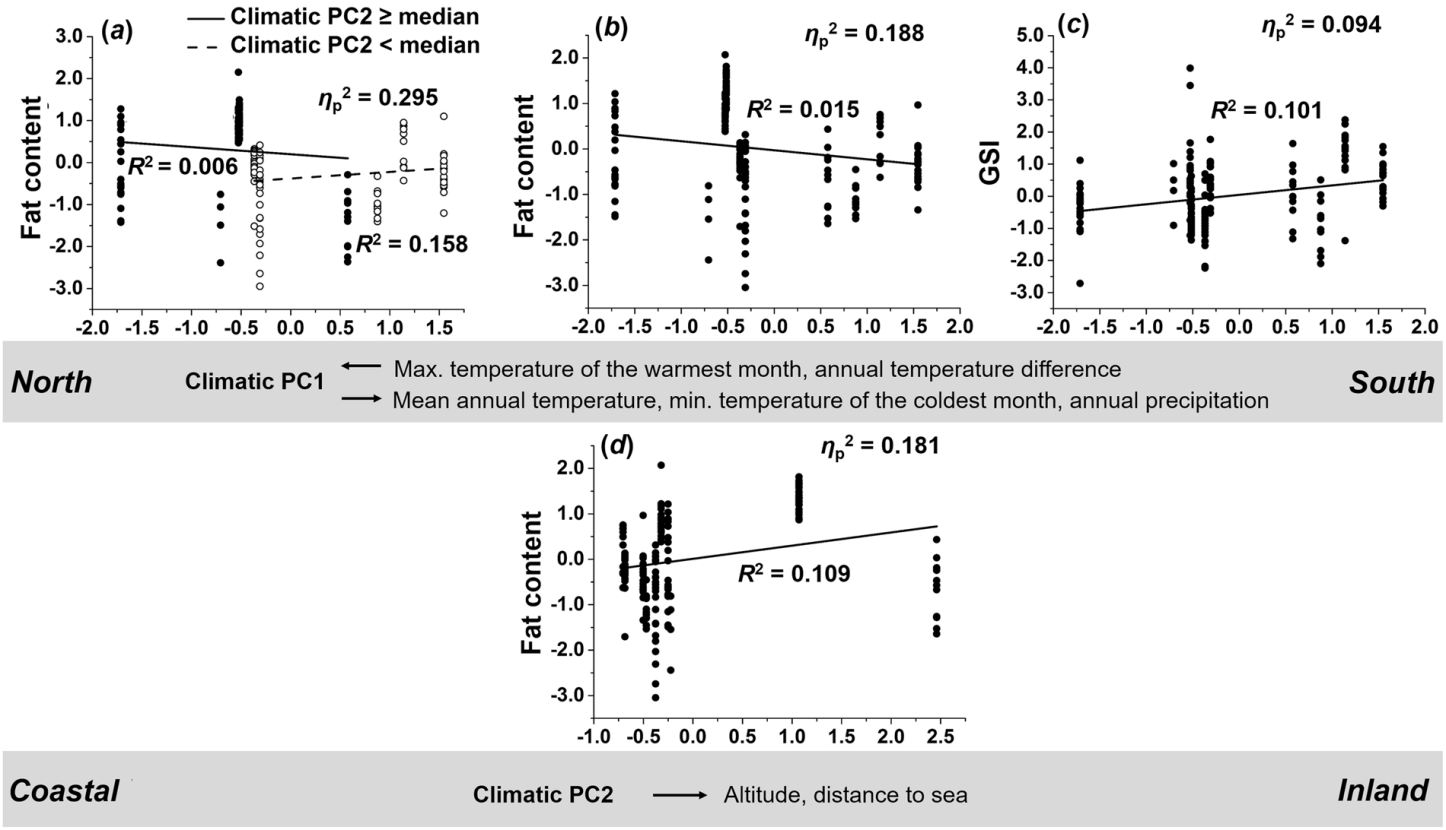
Climate-driven variation of male life-history traits. Depicted are the results of single-trait ANCOVAs (residuals, corrected for other model terms). (**a**) Males from inland sites (climatic PC2 ≥ median) showed a trend of decreasing body fat contents along climatic PC1 while coastal populations (climatic PC2 < median) showed the opposite pattern. (**b**) Overall, this led to a pattern where males decreased body fat content with increasing values of climatic PC1. (**c**) The gonadosomatic index (GSI) increased with increasing values of climatic PC1. (**d**) Furthermore, increasing values of climate-related PC2 resulted in increased body fat content.

Male life-history traits were affected by both climate-related PCs and their interaction (Table 2c for MANCOVA results; descriptive statistics of life-history data can be found in Table S4a). The interaction term (PC1 × PC2) showed the strongest effect (*η*_p_^2^ = 0.327), followed by PC1 (*η*_p_^2^ = 0.290) and PC2 (*η*_p_^2^ = 0.210). Below, we describe significant model terms from *post-hoc* ANCOVAs on all life-history traits (Table S5a), which are visualized in Fig. 5.

**Climate-related PC1** × **PC2:** The strongest interaction effect was seen on male fat content (*η*_p_^2^ = 0.295). This result reflects that males from inland populations tended to have less body fat towards the south (*R*^2^ = 0.006) while the trend was reversed in coastal populations (*R*^2^ = 0.158; Fig. 5a).

**Climate-related PC1:** Climate-related PC1 had the strongest effect on body fat content (*η*_p_^2^ = 0.188). This result can be interpreted as males overall showing decreasing body fat contents towards the south (*R*^2^ = 0.015; Fig. 5b). The weaker effect on the gonadosomatic index (GSI; *η*_p_^2^ = 0.094) suggests that males from southern populations exhibited an increased investment in reproductive tissues (*R*^2^ = 0.101; Fig. 5c).

**Climate-related PC2:** We found a significant effect of climate-related PC2 on body fat content (*η*_p_^2^ = 0.181), reflecting that males had increased body fat contents from coastal towards inland sites (*R*^2^ = 0.109; Fig. 5d).

### Females

**Figure 6:**
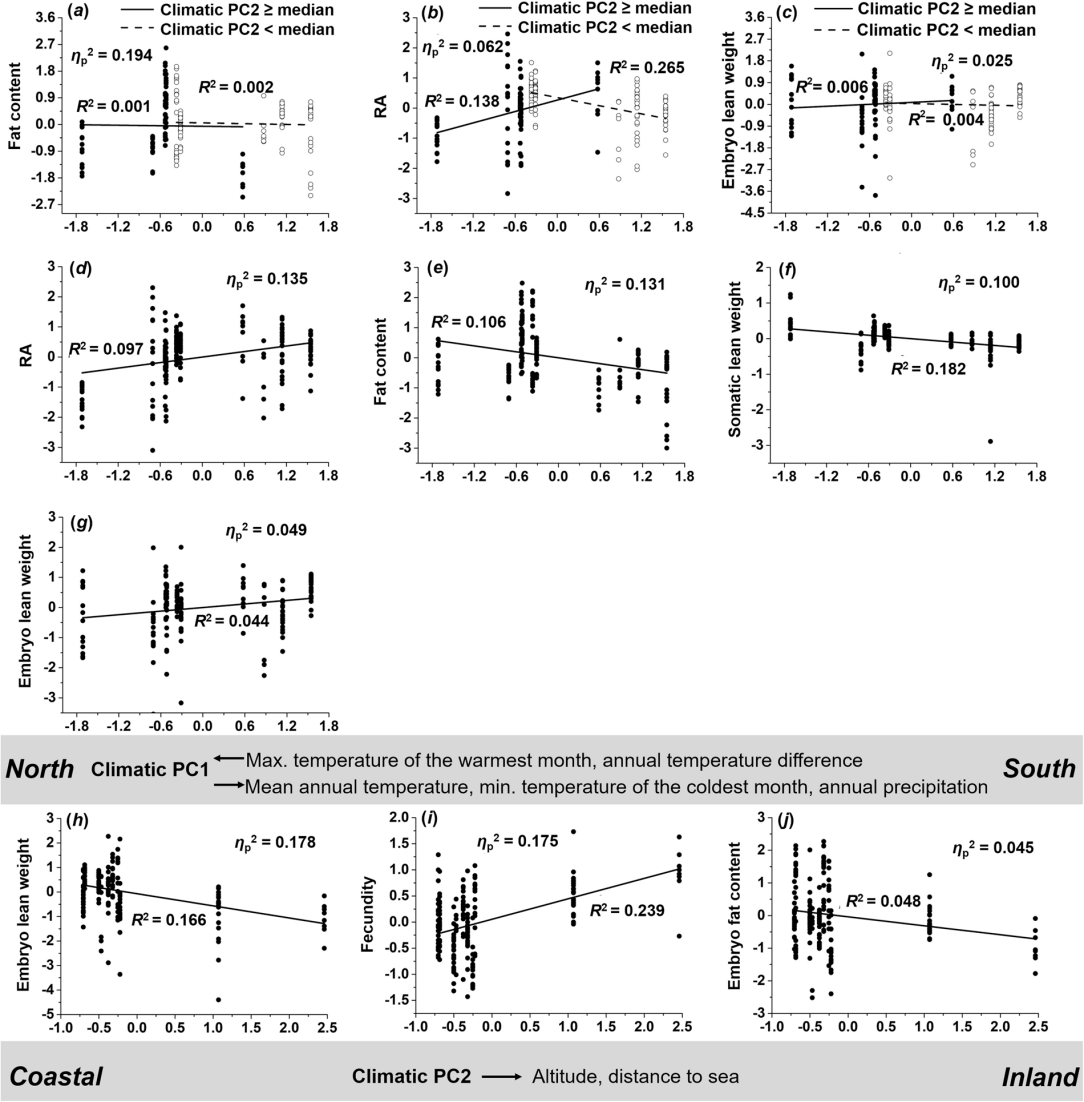
Variation in female life histories along climatic gradients. Depicted are the results of *post-hoc* ANCOVAs (residuals, corrected for other model terms). (**a**) As climate-related PC1 increases, body fat content showed a slight decrease in female *G. affinis*, while the trends differed slightly between coastal and inland populations. Females from inland populations increased (**b**) RA towards the south, while the trend was reversed in coastal populations. (**c**) A similar (but weak) trend was observed for embryo lean weight. Overall, this led to a pattern where females increased (**d**) RA and (**h**) embryo lean weight, but decreased (**e**) body fat content and (**g**) somatic lean weight with increasing values of climatic PC1. With increasing values of climate-related PC2, broods consisted of (**i**) more but (**h** and **j**) smaller embryos.

MANCOVA on female life-history traits found both climate-related PCs and their interaction to have significant effects (Table 2d; see also Table S4b for details). The interaction term had the strongest effect (*η*_p_^2^ = 0.332), followed by the main effects of climate-related PC1 (*η*_p_^2^ = 0.331) and PC2 (*η*_p_^2^ = 0.287). Significant model terms from *post-hoc* ANCOVAs on all life-history traits (Table S5b) are visualized in Fig. 6.

**Climate-related PC1** × **PC2:** Body fat content (*η*_p_^2^ = 0.194) and embryo lean weight (*η*_p_^2^ = 0.025) showed weak trends from north to south, and the trends differed slightly between coastal and inland populations (Fig. 6a and c). The interaction effect on reproductive allocation (RA; *η*_p_^2^ = 0.062) suggests that females from coastal populations decreased reproductive investment towards the south (*R*^2^ = 0.265), while the trend was reversed in inland populations (*R*^2^ = 0.138; Fig. 6b).

**Climate-related PC1:** Climate-related PC1 had an effect on RA (*η*_p_^2^ = 0.135), body fat content (*η*_p_^2^ = 0.131), somatic lean weight (*η*_p_^2^ = 0.100), and embryo lean weight (*η*_p_^2^ = 0.049), reflecting that females from southern sites showed elevated RA (*R*^2^ = 0.097; Fig. 6d), decreased body fat content (*R*^2^ = 0.106; Fig. 6e), decreased somatic lean weight (*R*^2^ = 0.182; Fig. 6f), and increased embryo lean weight (*R*^2^ = 0.044; Fig. 6g). Altogether, these results suggest that females from southern sites showed higher investment into reproduction (along with bigger embryos) than females from northern sites.

**Climate-related PC2:** Strong effects of climate-related PC2 were detected in the case of embryo lean weight (*η*_p_^2^ = 0.178) and fecundity (*η*_p_^2^ = 0.175), while a weak effect was found for embryo fat content (*η*_p_^2^ = 0.045). This reflects that females from inland sites had more (*R*^2^ = 0.239; Fig. 6i) but smaller embryos (*R*^2^ = 0.166; Fig. 6h) with a lower fat content (*R*^2^ = 0.048; Fig. 6j) than females from coastal populations.

#### Body shape variation

We digitized geometric landmarks and conducted Procrustes fits to extract geometric information. A factor reduction procedure was performed to reduce data dimensionality, and ten morphology-related PCs were retained for both males (accounting for 88.31% of the total morphological variance) and females (88.81%). We conducted similar analytical MANCOVAs and subsequently ran *post-hoc* ANCOVAs on single PCs as outlined above.

### Males

**Figure 7:**
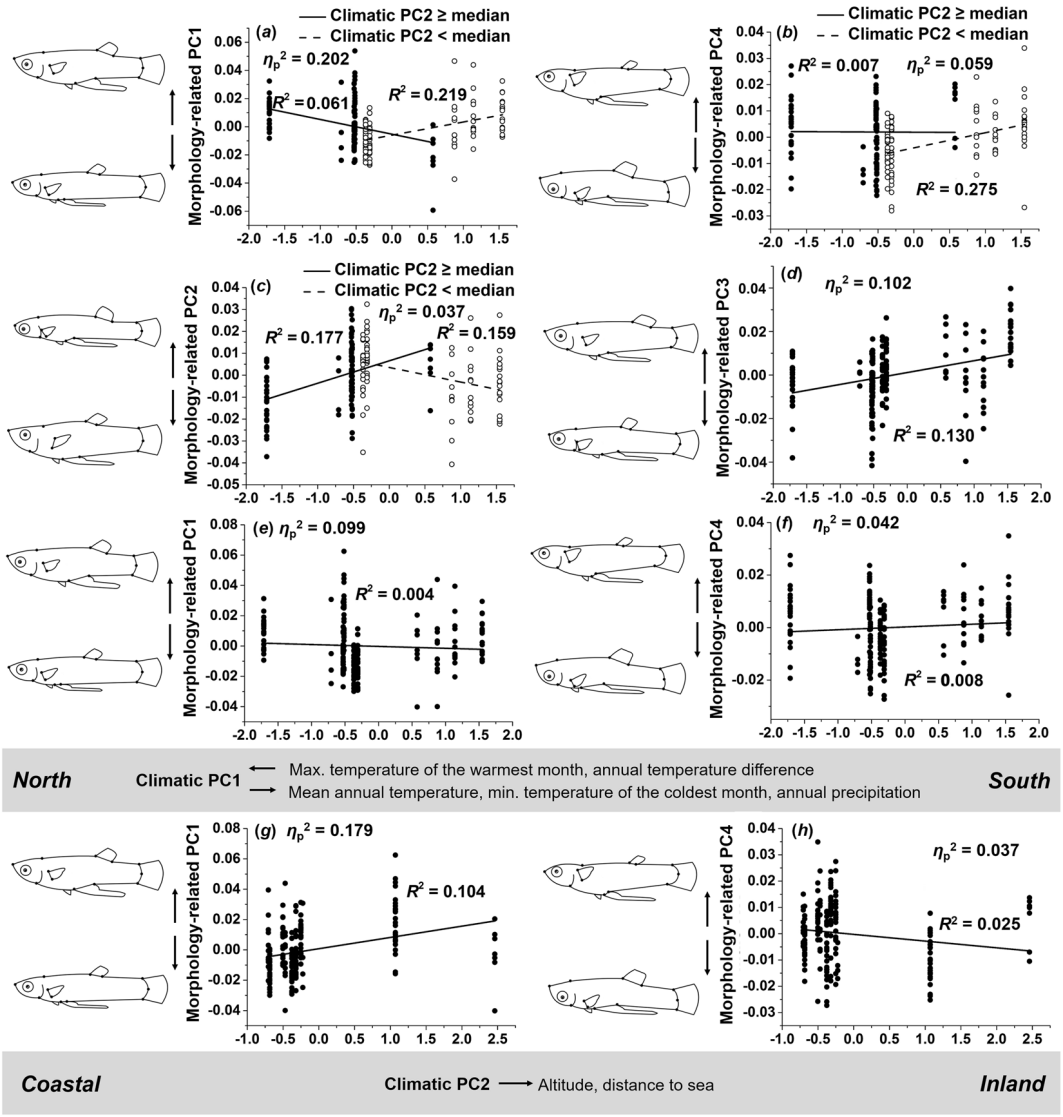
Variation of male body shape along climatic gradients. Increasing values of climatic PC1 (north to south) were associated with (**a**) more slender bodies, longer caudal peduncles and a more anterior gonopodium position in inland populations (climatic PC2 ≥ median) but a reversed trend in coastal populations (climatic PC2 < median), (**b**) more upward-oriented pectoral and caudal fins in coastal populations, which was not detected in inland populations, and (**c**) more slender bodies, smaller heads, and decreased peduncle lengths in inland populations, while the trend was reversed in coastal populations, (**d**) more slender bodies and increased caudal peduncle lengths across sites, (**e)** more slender bodies, longer caudal peduncles and a more anterior gonopodium, and (**f**) more upward-oriented pectoral and caudal fins. As climatic PC2 increases, (**g**) males showed deeper bodies, shorter caudal peduncles and a more posterior gonopodium, and (**h**) their pectoral and caudal fins had a lower position.

Our MANCOVA on male body shape found the interaction term to explain most of the variance (*η*_p_^2^ = 0.363), followed by the main effects of climate-related PC1 (*η*_p_^2^ = 0.361) and climate-related PC2 (*η*_p_^2^ = 0.320; Table 2e). Significant effects of ANCOVAs using the ten morphological PCs as dependent variables (Table S5c) are presented below and visualized in Fig. 7.

**Climate-related PC1** × **PC2:** The interaction term had the strongest effect on morphology-related PC1 (*η*_p_^2^ = 0.202). Males from inland populations (climate-related PC2 ≥ median) had more slender bodies, longer caudal peduncles and more anteriorly positioned gonopodia towards the south (i.e., with increasing values of climate-related PC1; *R*^2^ = 0.061), while the opposite trend was observed in coastal populations (climate-related PC2 < median; *R*^2^ = 0.219; Fig. 7a). A weaker effect was found for PC4 (*η*_p_^2^ = 0.059), reflecting that while males from inland populations showed only minimal variation (*R*^2^ = 0.007), males from coastal populations had more upward-oriented pectoral and caudal fins towards the south (*R*^2^ = 0.275; Fig. 7b). The weak effect on morphology-related PC2 (*η*_p_^2^ = 0.037) can be interpreted as inland populations showing more slender bodies, smaller heads and decreased peduncle lengths towards the south (*R*^2^ = 0.177) while this trend was reversed in coastal populations (*R*^2^ = 0.159; Fig. 7c).

**Climate-related PC1:** We found the strongest effect of climatic PC1 in the form of increasing values of morphology-related PC3 (*η*_p_^2^ = 0.102; *R*^2^ = 0.130). This reflects that males had more slender bodies and increased caudal peduncle lengths from northern towards southern sampling sites (Fig. 7d). A similar—but much weaker—trend was found for morphology-related PC1 (*η*_p_^2^ = 0.099), which suggests that males also possessed more anteriorly positioned gonopodia towards the south (*R*^2^ = 0.004; Fig. 7e). Male PC4 increased marginally along climatic PC1 (*η*_p_^2^ = 0.042), suggesting that males showed slightly more upward-oriented (i.e., more dorsally-positioned) pectoral and caudal fins towards the south (*R*^2^ = 0.008; Fig. 7f).

**Climate-related PC2:** We found a significant effect of climate-related PC2 on morphology-related PC1 (*η*_p_^2^ = 0.179), suggesting that males had deeper bodies and shorter caudal peduncles in inland populations (*R*^2^ = 0.104; Fig. 7g). A much weaker effect was seen for PC4 (*η*_p_^2^ = 0.037), and males from inland sites had slightly lower pectoral and caudal fin positions (*R*^2^ = 0.025; Fig. 7h).

### Females

**Figure 8:**
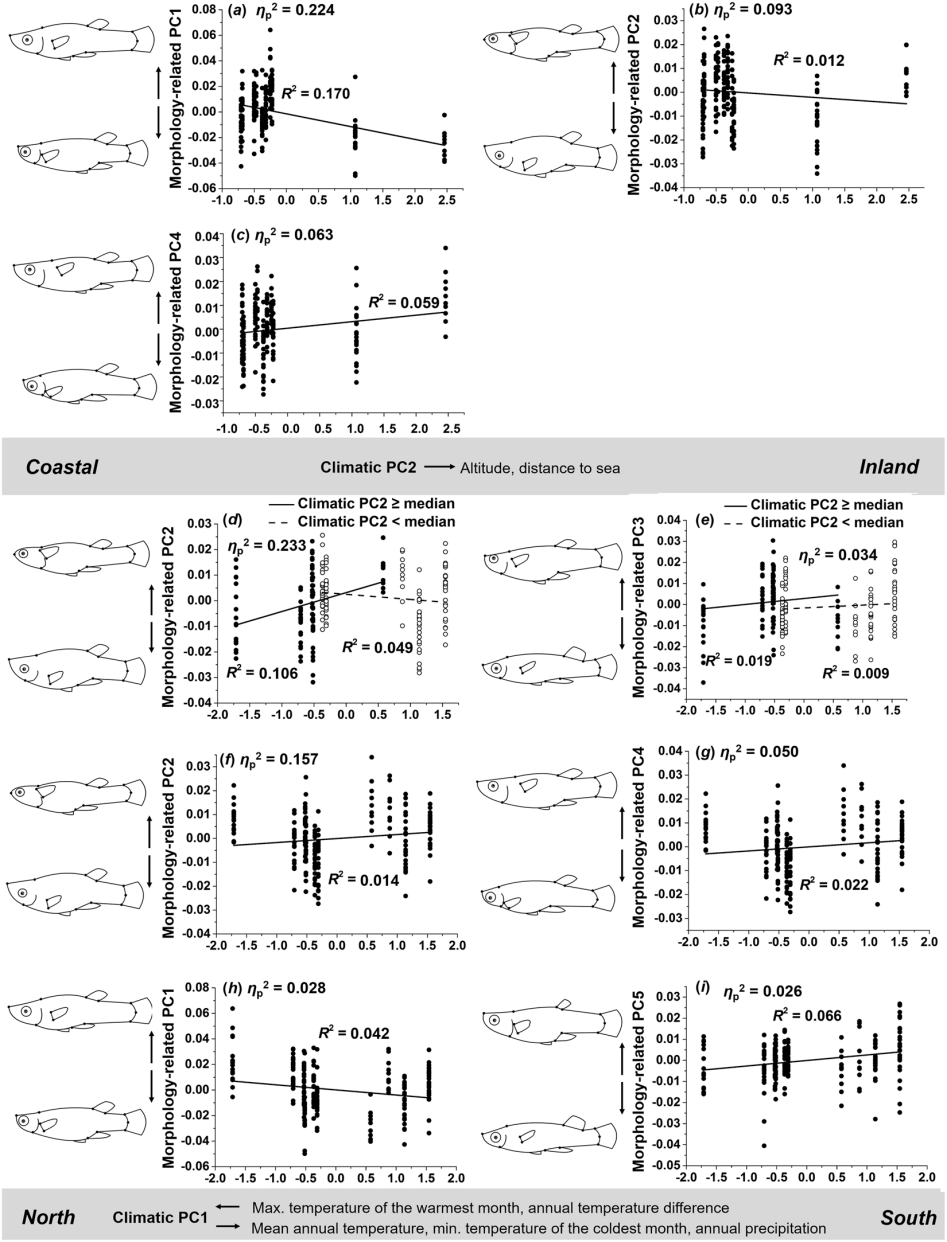
Variation in female body shape along climatic gradients. Increasing values of climatic PC2 resulted in (**a**) deeper bodies, smaller heads, more anterior pectoral fins and shorter caudal peduncles, (**b**) deeper bodies, bigger heads, more downward-oriented pectoral fins and caudal peduncles, and (**c**) bigger heads, more upward-positioned pectoral fins and shorter and more upward-oriented caudal peduncles. As values of climatic PC1 increased, female *G. affinis* showed (**d**) more slender bodies, smaller heads, and more upward-oriented positions of the pectoral and caudal fins in inland populations (climatic PC2 ≥ median), while the trend was reversed in coastal populations (climatic PC2 < median), (**e**) a trend towards deeper bodies, smaller heads, more upward-oriented pectoral and caudal fin in inland populations, while the trend was only weak in coastal populations, (**f**) more slender bodies, smaller heads, and more upward-oriented pectoral and caudal fins, (**g**) bigger heads, more upward-positioned pectoral fins and shorter and more upward-oriented caudal peduncles, (**h**) a trend towards deeper bodies, smaller heads, more anterior pectoral fin and shorter caudal peduncles, and (**i**) deeper bodies, bigger heads, and more anterior pectoral fins and longer caudal peduncles.

Our MANCOVA revealed that female morphology was significantly affected by climate-related PC2 (*η*_p_^2^ = 0.384), the interaction term of both climate-related PCs (*η*_p_^2^ = 0.330), and the main effect of climate-related PC1 (*η*_p_^2^ = 0.311; Table 2f). Significant effects of single-trait ANCOVAs on all ten morphology-related PCs (Table S5d) are visualized in Fig. 8.

**Climate-related PC2:** When comparing coastal and inland sites (i.e., increasing values of PC2), morphology-related PC1 decreased (*η*_p_^2^ = 0.224), reflecting that females from inland populations had deeper bodies, smaller heads, more anteriorly positioned pectoral fin, and shorter caudal peduncles (*R*^2^ = 0.170; Fig. 8a). Morphology-related PC2 decreased along climatic PC2 (*η*_p_^2^ = 0.093), which indicates that females from inland populations had deeper bodies, bigger heads, and more downward-oriented (i.e., ventrally-positioned) pectoral fins and caudal peduncles (*R*^2^ = 0.012; Fig. 8b). The weak effect on morphology-related PC4 (*η*_p_^2^ = 0.026) reflects a trend for females from inland populations to show bigger heads, more upward-positioned pectoral fins, and shorter and more upward-oriented caudal peduncles (*R*^2^ = 0.059; Fig. 8c).

**Climate-related PC1** × **PC2:** The strongest interaction effect was detected in the case of morphology-related PC2 (*η*^2^ = 0.233). Females from inland populations developed more slender bodies, smaller heads, and more upward-oriented pectoral and caudal fins towards the south (*R*^2^ = 0.106), while a tendency towards a reversed pattern was seen in coastal populations (*R*^2^ = 0.049; Fig. 8d). The weak interaction effect in case of morphology-related PC3 (*η*^2^ = 0.034) suggests that the overall effect of females having deeper bodies, smaller heads, as well as more upward-oriented positions of the pectoral and caudal fins was stronger in inland (*R*^2^ = 0.019) than coastal populations (*R*^2^ = 0.009; Fig. 8e).

**Climate-related PC1:** Climate-related PC1 had the strongest effect on morphology-related PC2 (*η*_p_^2^ = 0.157), reflecting that females from southern populations had more slender bodies, smaller heads, and more upward-oriented positions of the pectoral and caudal fins (*R*^2^ = 0.014; Fig. 8f). A weaker effect was detected in the case of morphology-related PC4 (*η*_p_^2^ = 0.050). This effect suggests that females had bigger heads, more upward-positioned pectoral fins, as well as shorter and more upward-oriented caudal peduncles towards the south (*R*^2^ = 0.022; Fig. 8g). We detected weak effects of climate-related PC1 on morphology-related PC1 (*η*^2^ = 0.028) and PC5 (*η*^2^ = 0.026). Morphology-related PC1 decreased as climate-related PC1 increased, suggesting that females from southern sites had slightly deeper bodies, smaller heads, more anteriorly-positioned pectoral fins, and shorter caudal peduncles (*R*^2^ = 0.042; Fig. 8h). Increasing values of morphology-related PC5 along climate-related PC1 suggests that females showed somewhat deeper bodies, bigger heads, more anteriorly-positioned pectoral fins, and longer caudal peduncles towards southern sites (*R*^2^ = 0.066; Fig. 8i).

#### Gonopodium morphology and length

Morphological information on gonopodium tip structures was collected using similar Procrustes analyses and subsequent PCA as described for the body shape analyses. We retained nine gonopodium morphology-related PCs (accounting for 89.93% of the total variance) and used them as dependent variables in MANCOVA and *post-hoc* ANCOVAs. Gonopodium length was collected during the assessment of body shape information (see Methods) and was subjected to an ANCOVA using SL and the two climate-related PCs as covariates.

### Gonopodium morphology

**Figure 9:**
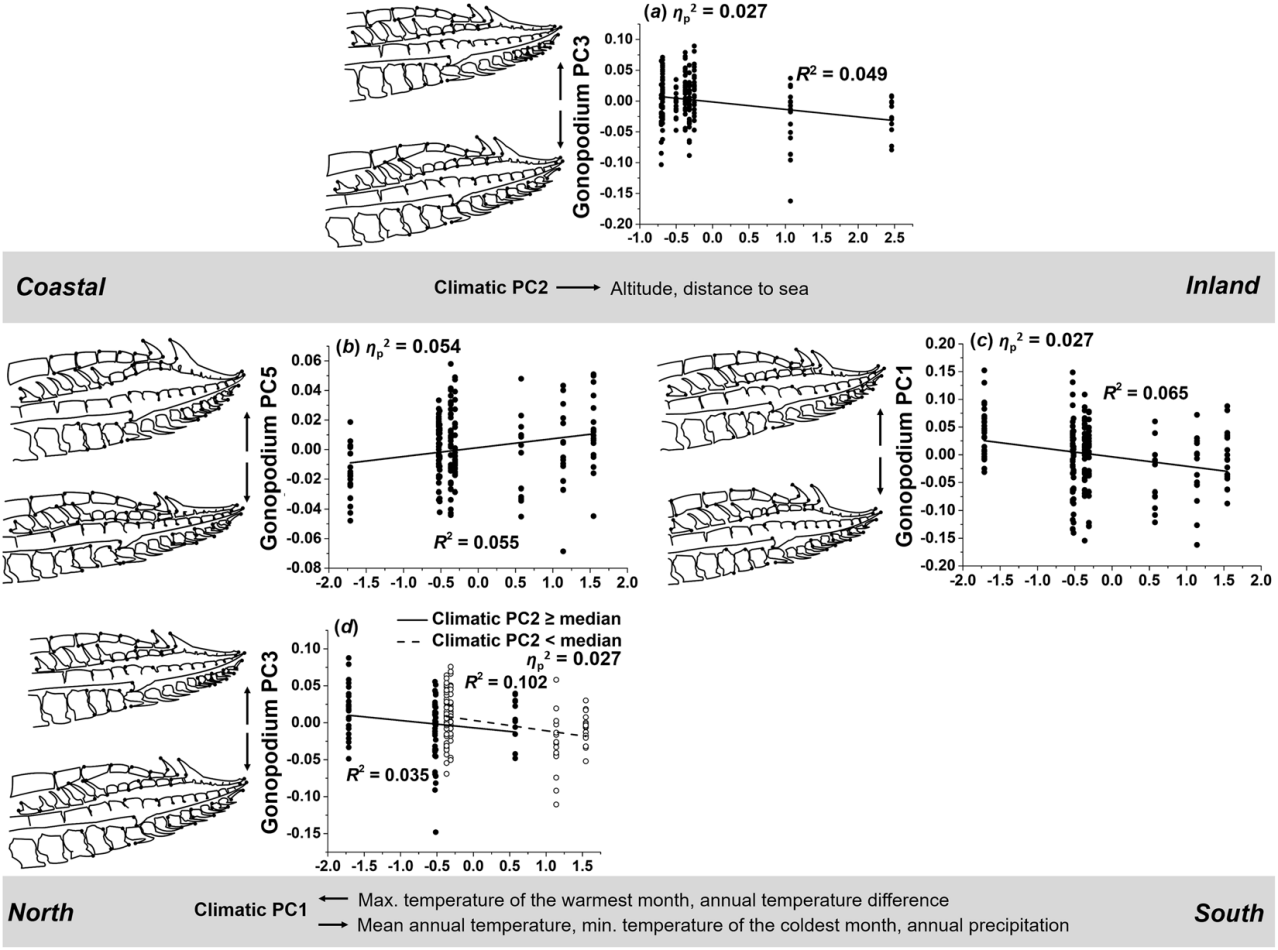
Variation in gonopodium morphology along climatic gradients. As climatic PC2 increases (i.e., in inland populations), males tended to have (**a**) a deeper gonopodium with looser spines, along with longer gonopodium tips. Increasing values of PC1 resulted in (**b**) a deeper gonopodium, larger hooks and a wider cavity between fin rays 4a and 4p, (**c**) slightly shortened gonopodium tips, and (**d**) a deeper gonopodium with looser spines (the latter effect differed slightly between coastal and inland populations).

Our MANCOVA on PCs capturing divergence in gonopodium tip shape found significant effects of both climate-related PCs and their interaction effect (Table 2g), whereby effect sizes decreased from climate-related PC2 (*η*_p_^2^ = 0.164) over PC1 (*η*_p_^2^ = 0.133) to the interaction term (PC1 × PC2; *η*_p_^2^ = 0.120). Significant effects from *post-hoc* ANCOVAs (Table S5e) are visualized in Fig. 9.

**Climate-related PC2:** We found a weak effect of climate-related PC2 on gonopodium morphology-related PC3 (*η*_p_^2^ = 0.027), reflecting that males from inland populations had slightly deeper and longer gonopodium tips, with a looser arrangement of the spine-like structures at the tip of anal fin ray 3, than males from coastal sites (*R*^2^ = 0.049; Fig. 9a).

**Climate-related PC1:** The strongest effect of climate-related PC1 was seen in the case of gonopodium morphology-related PC5 (*η*_p_^2^ = 0.054). This result suggests that males had a deeper gonopodium tip, larger hooks and a wider cavity between anal fin rays 4a and 4p towards southern sites (*R*^2^ = 0.055; Fig. 9b). The weak effect on gonopodium morphology-related PC1 (*η*_p_^2^ = 0.027) reflects that males from southern populations tended to have shorter gonopodium tips (*R*^2^ = 0.065; Fig. 9c).

**Climate-related PC1** × **PC2:** We found a weak interaction effect on gonopodium morphology-related PC3 (*η*_p_^2^ = 0.027), which suggests that males from inland populations had a slightly deeper gonopodium tip, while the spine-like structures at the tip of anal fin ray 3 were less tightly aligned (*R*^2^ = 0.035; Fig. 9d).

### Gonopodium length

**Figure 10:**
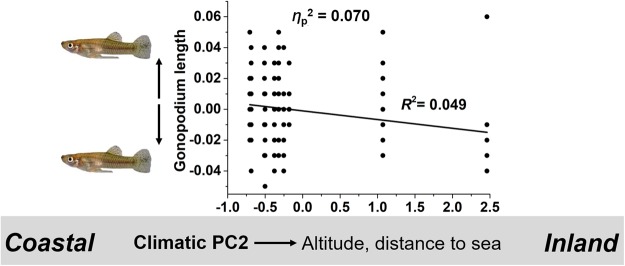
Variation in gonopodium length along climate-related PC2. Visualization of the main effect of climatic PC2 on gonopodium length (residuals, corrected for other model terms; see Table 2h). As climatic PC2 increased (i.e., towards inland populations), males tended to have a shorter gonopodium.

Our ANCOVA on gonopodium length detected a significant effect of climate-related PC2 (*η*_p_^2^ = 0.070), reflecting that males from inland populations had a shorter gonopodium (*R*^2^ = 0.049; Fig. 10).

## Discussion

The role played by natural selection in driving phenotypic diversification along climatic gradients has been acknowledged by a multitude of studies^3,85,86^. Diversification of traits that are prime targets of sexual selection, however, has received far less attention^87,88^. We used invasive populations of Western mosquitofish (*G. affinis*) to investigate potential divergence of phenotypic traits known to be under different forms of selection. Population genetic analyses based on neutral markers found the ten examined populations to form two divergent clusters, confirming that the species was repeatedly introduced to mainland China^56^. Nevertheless, we found a signature of isolation-by-distance, which could imply ongoing gene-flow between populations. Phenotypic variation among populations did not follow a pattern reflecting the two population genetic clusters; rather we found gradual variation of various trait suites along climatic gradients (Figs 3–10, S1–5).

Our results confirm the prediction that traits under sexual selection can diverge systematically along climatic gradients. Still, the strongest effects were observed for body shape diversification, followed by life-history traits, both of which are thought to be prime targets of natural selection^14,85,89–91^. The shape of the distal tip of the male copulatory organ (the gonopodium—a sexually selected trait^64,92–94^) and gonopodium length showed weaker variation along climatic gradients. Finally, male (but not female) body size showed a weak (but significant) signal congruent with Bergman’s rule^40,57^; i.e., males became larger towards higher latitudes and towards more coastal sites. Overall, males and females showed phenotypic divergence in response to different components of climatic variation (see summary results in Fig. 3), suggesting different evolutionary trajectories for both sexes^95–99^: males diverged especially along the latitudinal gradient (climatic PC1), while females diverged primarily along the longitudinal/continental gradient (climatic PC2). In the following, we will discuss the observed patterns of phenotypic variation in light of our *a priori* hypotheses. We will start with gonopodium morphology, such that the discussion of divergence of other trait suites will be informed by inferences regarding the contribution of systematic variation of sexual selection along climatic gradients.

### Gonopodium morphology and length

Climate-related variation in the morphology and length of the male intromittent organ, the gonopodium^92,93^, was mostly in support of our *a priori* predictions: males from southern populations possessed a wider gonopodium tip with increased armament compared to males from northern sites. Considering longitudinal variation, males had a longer gonopodium with shorter and more ‘condensed’ genital tips towards coastal sites. The morphology of the distal gonopodium tip affects insemination and fertilization success in poeciliid fishes^15,65,93^. For instance, male guppies (*P. reticulata*) with shorter distal tips but a longer overall gonopodium were more successful at achieving genital contact with females, and more sperm were recovered from the female gonoduct when females interacted with such males^100^. A longer gonopodium with a condensed and shorter genital tip in *G. affinis* males from coastal populations could thus be a result of fierce mating competition in more stable (coastal) environments. An additional (not mutually exclusive) explanation would be that female choice for elongated gonopodia^64,101^ is stronger in coastal populations, for example, as females may become choosier when mate availability increases^102–105^.

We found a wider cavity between fin rays 4a and 4p and larger gonopodium hooks towards the south (i.e., along climatic PC1), which could again reflect fiercer mate competition in southern populations, where population densities tend to be high throughout the year^106,107^. Hooks may serve as a holdfast during mating^108^ and species with larger hooks tend to have longer copulations, which enhances insemination or post-insemination fertilization success under strong intrasexual competition^106–109^. Another, not necessarily mutually exclusive explanation assumes larger hooks to injure the female gonoduct, which could prevent females from remating^108,110^. The function of deeper genital tips, along with a widened cavity between fin rays 4a and 4p, remains elusive. Generally, structures should be positively selected that increase the amount of sperm bundles (spermatozeugmata) transferred per copulation, as increased sperm transfer is commonly observed in poeciliid males under heightened mate competition^111,112^. Overall then, we found that sexual selection arising from male mate competition (and possibly female choice^113,114^) appears to have left its footprints on phenotypic divergence along both climatic gradients considered herein, and we argue that systematic covariation of population ecological parameters with climatic conditions alters the selective landscapes along those gradients.

### Life-history variation

We predicted that higher extrinsic mortality rates at higher latitudes would select for increased reproductive investment, but our results did not confirm our *a priori* predictions. Overall, male *G*. *affinis* from inland populations showed reduced fat content, while coastal populations showed increased fat content towards southern populations. We also found males to exhibit increased gonosomatic indices (GSI) and decreased somatic lean weights in southern populations, while females showed increased reproductive allocation (RA), decreased body fat contents, decreased somatic lean weights, and increased embryo lean weights. Moreover, males had increased fat contents, while females showed lower embryo fat contents and produced more but smaller embryos in inland populations.

Early life-history evolution models considered extrinsic mortality as the main driver of life-history divergence^14,115^, but recent studies emphasize the roles played by population densities, resource availability and competition^116,117^. As suggested to explain the results from other parts of our study, we argue that population densities—and thus, altered levels of competition (both for resources and mates)—may be important agents shaping life-history trait divergence along climatic gradients. Sperm competition intensifies as a function of lower overwinter mortality under benign (southern) conditions^118,119^. A higher GSI may allow for increased sperm production under these circumstances^120,121^. However, further studies are required to fully elucidate the impact of population densities on the observed life-history divergence. Likewise, female poeciliids typically produce fewer and bigger offspring that are more competitive under fierce resource competition^70,121–123^. Decreased fat content towards southern populations in both sexes could thus reflect a trade-off between reproductive investment and investment into somatic maintenance in the face of strong resource competition^117^. We are lacking a clear explanation with respect to the observed divergence of somatic lean weight, but increased lean weight could reflect an adaptation to enhance growth and reproduction during the shorter growing seasons in northern latitudes^57^.

Females (from northern populations) and males showing increased fat content in inland populations may be indicative of relaxed resource competition towards inland sites. The pattern observed in females from southern sites, however, matches predation-driven patterns described for other poeciliids, with lower fat content, and more but smaller offspring being produced in inland populations^14,124,125^. Large body size in poeciliids can be accompanied by an increased risk of falling victim to predation^126,127^, and avian predation exerts strong selection on body size at least in natural *G. affinis* populations^128^. However, we are currently lacking empirical data on potential variation in avian predation along the climatic gradients examined here.

### Body shape and size variation

We hypothesized that body shape divergence would primarily follow patterns observed for life-history diversification, with enlarged abdominal cavities, anteriorly positioned pectoral fins and smaller heads at higher latitudes. We found patterns of divergence to be seemingly congruent with our predictions, but notably, the pattern was more clear-cut for males than for females. This sheds doubt on our initial hypothesis that body shape would evolve as an indirect consequence of life-history divergence, in which case females should show the strongest body shape divergence. We further predicted increased body size at higher latitudes and inland sites because large-bodied individuals have a higher survival rate in harsh and fluctuating environments. Our predictions were met along the latitudinal gradient (climatic PC1) but reversed along the longitudinal gradient (PC2), and the pattern was only observed in males.

Previous studies on freshwater fishes identified several agents of natural selection to affect body shape and size, including flow regime^129^, resource availability^130,131^, and predation risk^14,89–91,132^. We argue that variation in both body shape and size of male *G. affinis* in our study system is primarily driven by temperature regimes. Low overwinter temperatures at higher latitudes select for endurable individuals with larger body size^133^ and larger fat reserves^134^. Larger fat stores could indeed explain enlarged abdominal cavities of male *G. affinis* from northern populations. Mosquitofish males are much smaller, on average, than females^135^, and so males may be under stronger selection for increased body size (and more compact shape) at higher latitudes. Moreover, we suggested increased intrasexual competition in southern populations, but also higher predation pressure in the south could translate into more forced copulations^10,127^. Enlarged caudal regions, smaller heads, and a more elongated body are traits that improve unsteady swimming^87,136^, which is also important during coercive mating. Moreover, small-bodied males can approach females in the blind portion of their visual field, thus preventing females from fleeing, and have a better maneuverability than large-bodied ones^137–139^.

Predation selects for early maturity and smaller adult body size in numerous poeciliid species^140^ like *Poecilia reticulata*^14^, *P. vivipara*^141^, *Brachyrhaphis episcopi*^142^ or *Phalloceros harpagos*^143^. The smaller body size of males from inland populations could again point towards a role for increased predation risk along the longitudinal gradient (see above), for which we do not currently have empirical evidence at hand. As poeciliid females tend to show a preference for large-bodied males^64,144–148^, our results are also congruent with a more important role for female choice in coastal populations, which we have previously discussed to explain patterns of divergence in gonopodium length (see above).

Unlike male body shape, female body shape showed most variation along the longitudinal and to a lesser extent the latitudinal gradient, and the observed patterns could indeed reflect covariation with life-history traits. According to life-history theory, harsh and fluctuating environments—as found in inland regions—select for increased reproductive effort^62,149^. Enlarged abdominal regions could thus be a result of larger broods, as supported by our life-history analysis, and enlarged abdominal regions would bring about relatively smaller heads and more anteriorly positioned pectoral fins. Females showed a more slender overall body shape along the latitudinal gradient, which could simply be a consequence of lower body fat reserves towards the south. Divergence of other traits, including decreased eye and head sizes, remains elusive.

## Conclusion

Biological invasions have received considerable attention in conservation biology^150–152^ as several invasive species threaten native species, communities, and ecosystem functioning^153–155^. At the same time, invasive species provide an excellent opportunity to investigate adaptive intraspecific diversification on a contemporary scale^156,157^. Mosquitofish were introduced to mainland China ~90 years ago^158,159^, equaling between 270 and 360 generations in southern regions^53,160^. Our current study suggests that several traits under natural selection (aspects of body shape, life-history traits) diverge more strongly along climatic gradients in the species’ invasive distribution range than primarily sexually selected traits (gonopodium tip contours). It will be exciting for future studies to ask if the relative strength of divergence of different trait suites may change with time, e.g., if divergent sexual selection is comparatively weak but its action continues, while some of the naturally selected traits were immediately driven to their phenotypic optimum^161,162^. Future studies should also address the question of whether and to what extent trait divergence is caused by evolutionary divergence or phenotypic plasticity, and formulate testable predictions regarding the roles of adaptive and non-adaptive plasticity for evolutionary divergence. Adaptive phenotypic plasticity is thought to slow down evolutionary rates, while non-adaptive plasticity may accelerate genetic evolution^163–165^.

Our present study supports the idea that Bergmann’s rule—originally formulated for endotherms^32^—can be expended and used more broadly to explain body size variation in some groups of ectotherms^38–40,49^. We argue that patterns congruent with Bergman’s rule may be driven primarily by differential overwinter survival^40,166–168^. A caveat of our study is that large parts of our discussion are based on the assumption that population densities (and thus, competition) vary systematically along the climatic gradients considered herein; yet, some of the patterns also point towards divergent selection from predation. Predation drives various forms of phenotypic diversification in livebearing fishes; e.g., predation differs systematically in streams inhabited by Trinidadian guppies (upstream sites are characterized by low predation and downstream sites by high predation^14,169^). Future studies will need to assess additional ecological factors covarying with the climatic gradients considered here, with a special focus on biotic selection factors.

## Methods

The current study does not include experiments involving living animals. All experimental procedures were approved by the Animal Welfare commissioner at the Department of Animal Science of the College of Animal Science and Technology, Northwest A&F University. All experiments were performed in accordance with relevant guidelines in China (Standards for the investigation of reservoir fishery resources, SL 167–2014).

### Sample collection and climatic data

China harbors an array of different climate zones, from tropical climates in the south to cold temperate climates in northern parts^170^. *Gambusia affinis* was reported primarily along the Yangtze River^56^, with most of its catchment being situated in subtropical parts of China^171,172^. We collected *G. affinis* during the reproductive season (between April and September 2016) at 10 sampling sites across the species’ invasive range in China^56^ using dip nets (2 mm mesh size). Collection sites were stagnant or slow-flowing water bodies with dense riparian vegetation (Fig. 1d–f). Upon capture, all specimens were sacrificed with an overdose of clove oil. We preserved specimens in 96% ethanol and transferred them to our laboratory at Northwest A&F University for subsequent analyses.

We downloaded climatic data (from 1981–2012) from the Chinese meteorological data network (http://data.cma.cn/) at 0.5° × 0.5° resolution. We included (1) mean annual temperatures, (2) maximum temperatures of the warmest month, (3) minimum temperatures of the coldest month, (4) annual temperature differences (by subtracting the minimum monthly temperature from the maximum monthly temperature), and (5) annual precipitation to provide site-specific climatic information (see Table 3 for details). (6) Altitude and (7) distance to the sea were obtained from Google Earth (http://earth.google.com/). We condensed those variables by means of a PCA, resulting in two PCs with eigenvalues >1 that explained 85.54% of the variation (Table 1). PC1 described the gradient from northern towards southern sites (latitudinal variation), whereby northern sites showed lower mean annual temperatures, lower minimum temperatures of the coldest month, lower annual precipitation, higher maximum temperatures of the warmest month, and higher annual temperature differences. PC2 described gradual changes from coastal towards inland sites (longitudinal variation; Table 1).

**Table 3:** Variation of climatic and geographic parameters across 10 sampling sites from which invasive *G. affinis* were collected.

Population	Mean annual temperature [°C]	Maximum temperature of the warmest month [°C]	Minimum temperature of the coldest month [°C]	Annual temperature difference [°C]	Annual precipitation [mm]	Altitude [m]	Distance to the sea [km]
Baoding (BD)	13.3	41.6	−16.8	10.1	496.1	25	205
Ankang (AK)	15.7	41.3	−9.7	9.0	824.1	275	1,128
Nanjing (NJ)	15.9	40.0	−13.1	8.5	1,090.7	77	207
Chengdu (CD)	16.3	37.5	−4.6	7.4	855.7	485	1,682
Huzhou (HU)	16.3	39.2	−8.5	7.4	1,303.4	25	179
Hangzhou (HA)	17.0	40.3	−8.4	7.8	1,438.1	16	52
Lishui (LS)	18.4	43.2	−7.3	9.3	1,406.0	265	105
Xiamen (XM)	20.7	39.2	1.5	7.0	1,332.4	15	0
Chaozhou (CZ)	22.7	39.4	2.1	7.6	1,726.0	17	29
Beihai (BH)	22.9	37.1	2.6	6.5	1,775.2	22	0

Climatic data (from 1981–2010) were obtained from the Chinese meteorological data network (http://data.cma.cn/). Altitude and distance to the sea were obtained from Google earth (http://earth.google.com/).

### DNA extraction and microsatellite analyses

We included *n* = 100 males and *n* = 100 females in our population genetic analysis (ten males and ten females per population). We extracted whole genomic DNA from pectoral fin tissue using the EasyPure Genomic DNA Kit (Beijing TransGen Biotech, Beijing, China). Our analysis was based on 15 previously published nuclear microsatellite markers^78–80^. Primer dye-groups are listed in Table S6. We first amplified all markers separately. Each 5 µl reaction mix included 2.5 µl 2 × Taq MasterMix (CWBIO, Beijing, China), 0.4 µl primer mix, 1.3 µl RNase-free water, and 0.8 µl template DNA. Thermocycling conditions were as follows: initial denaturation at 94 °C for 3 min, followed by 35 cycles of 94 °C for 30 s (denaturation), 60 °C for 30 s (primer annealing), 72 °C for 30 s (elongation), and a final elongation step at 72 °C for 10 min. Before fragment length analysis, we mixed equal amounts of PCR products as follows: mix 1 (Gaaf11, Gaaf13, Gaaf16, Gafu1, Gafu3), mix 2 (Gaaf7, Gaaf9, Gaaf15, Gaaf22, Gafu2, Gafu6), and mix 3 (Gaaf10, Gafu4, Gafu7, Mf-13). Fragment sizes were scored manually after electrophoresis on an ABI3730 sequencer, using Liz500 as the internal size standard.

We used Arlequin 3.5^173^ to calculate expected (*H*_E_) and observed heterozygosity (*H*_O_), and to test for deviations from Hardy-Weinberg-Equilibrium. FSTAT v 2.9.3.2^174^ was used to calculate allelic richness (*A*). We further tested for null alleles at each locus using Micro-checker v 2.2.3^175^. Following the methods described in Chapuis and Estoup^176^, we used FreeNA to calculate unbiased *F*_ST_-values between populations while accounting for potential null alleles. To estimate the degree of isolation-by-distance among populations, we performed a Mantel test with pairwise *F*_ST_-values (calculated with FreeNA using the ENA correction) and linear geographic distances (obtained from Google Earth) using IBDWS v 3.23 (http://ibdws.sdsu.edu/ibdws/distances.html). We tested for evidence of genetic bottlenecks in each population separately using Bottleneck v 1.2.02^177^. We used Wilcoxon signed-rank tests to identify recently bottlenecked populations by comparing observed and expected numbers of loci with heterozygosity excess under three mutation models, the infinite allele model (IAM), stepwise mutation model (SMM), and two-phase model (TPM), respectively, as recommended by Luikart and Cornuet^178^.

We used STRUCTURE v 2.3.4^179^ to calculate individual assignment probabilities (*Q*-values) to varying numbers of genetically distinct clusters (*K*). For each value of *K* = 1–10, ten iterations were run using the admixture model with a burn-in period of 250,000 generations, followed by a sampling phase of 750,000 iterations. We detected the uppermost level of population differentiation with the method presented by Evanno *et al*.^180^ using the web-based tool STRUCTURE HARVESTER v 0.6.94. Furthermore, we calculated genetic distances^181^ (Nei’s *D*_A_) using Populations v 1.2.32 (http://bioinformatics.org/project/?group_id=84) and visualized a neighbor-joining tree using TreeView v 1.6.6^182^ (http://taxonomy.zoology.gla.ac.uk/rod/rod.html). The bootstrapping procedure implemented in Phylip v 3.695 (http://evolution.genetics.washington.edu/phylip.html) was used to evaluate the significance of tree nodes (based on allele frequencies, with 1,000 bootstrap replicates). Moreover, we analyzed genetic structure among populations by means of a principal coordinate analysis (PCoA) based on pairwise Nei’s using GenAlEx v 6.503^183,184^.

### Body size and life histories

We included *n* = 184 males and *n* = 191 females in the analysis of life-history traits (18 to 52 individuals per population). We measured standard lengths (SL) of each individual using digital calipers (accurate to the closest 0.01 mm). Maturity was assessed by inspecting the opened body cavity for developing ova (females) or mature testes (males). Afterwards, we removed all reproductive tissues and all developing embryos. We determined the stage of development and number of embryos (fecundity) for each female^185^. Somatic tissues, along with gonads or embryos, were then dried at 55 °C for 24 hours. To assess female and embryo body condition, dried samples were washed for at least six hours in petroleum ether to extract non-structural body fat and were then re-dried and re-weighed.

We thus assessed standard length (SL [mm]), somatic dry weight [mg], somatic lean weight [mg], and fat content [%] for both sexes, the GSI [%] for males, and fecundity (number of developing embryos), RA [%], embryo lean weight [mg], as well as embryo fat content [%] in case of females. Reproductive effort (i.e. GSI for males and RA for females) was calculated by dividing gonad dry weight (plus embryo dry weight in the case of females) by the sum of gonad (plus embryo) and somatic dry weights. We log_10_-transformed SL, somatic dry weight, and somatic lean weight, arcsine (square root)-transformed somatic fat content, GSI, RA and embryo fat content, and square root-transformed fecundity. *Z*-transformation was subsequently applied to all data to obtain unit-free data with equal variance.

To assess the extent of divergence along climatic gradients, we used MANCOVA using the two climate-related PCs (see above) as covariates. Throughout this study, we also included the interaction term of both climate-related covariates but removed it from the final models if not significant. In male life-history analyses, we further included SL as a covariate, while SL and the embryos’ developmental stage served as additional covariates in the case of females. We ran *post-hoc* ANCOVAs of the exact same structure as the final retained MANCOVA model, to identify the source(s) of variation in case of significant model terms. To evaluate the relative importance of each term, we estimated effect sizes by calculating Wilk’s partial eta squared (*η*_p_^2^)^85^. Furthermore, we report relative variance explained by model terms as the partial variance explained for a given term divided by the maximum partial variance in that model.

Generally, to visualize significant interaction effects, we split the data into inland (climate-related PC2 ≥ median) and coastal populations (PC2 < median) and depict variation along PC1 (latitudinal variation) for both cohorts. The alternative way of depicting variation along PC2, while splitting the data based on median values of PC1, is shown in Supplementary Figs S1 and S2.

### Geometric morphometrics

We included *n* = 191 males and *n* = 211 females in the analysis of body shape divergence (17 to 57 individuals per population). We took lateral photographs of alcohol-preserved individuals (left body side) that were placed in a paraffin-coated petri-dish alongside a piece of laminated scale grid paper using a Canon EOS 760D single lens reflex camera (CANON INC., Ota-Ku, Japan). Photos were loaded into tps format using tpsUtil software^186^, after which we digitized 13 landmarks and measured gonopodium length (in the case of males) using tpsDig2 v 2.26^187^ (Fig. 1b). Landmarks provided adequate coverage of the lateral body contour of mosquitofish^82,188^. To correct for bending effects, we applied the ‘Unbend specimens’ function in tpsUtil using landmarks 1 and 6, as well as two additional landmarks (14 and 15) that were removed from the final analysis (Fig. 1b). We then applied a full Procrustes fit procedure using the software MorphoJ^188^. This procedure superimposes shape coordinates in a linear tangent space and automatically excludes variation that is not caused by true shape-variation (i.e. translation, scaling and rotation effects). After extracting shape information, a factor reduction procedure was performed in MorphoJ to reduce data dimensionality. We retained ten morphology-related PCs for both males and females, which accounted for 88.31% (males) and 88.81% (females) of the total morphological variance, respectively.

Our main analytical MANCOVA used morphology-related PCs as dependent variables and log_10_-transformed centroid size along with the two climate-related PCs (see above) as covariates. Again, *post-hoc* ANCOVAs on single PCs were conducted as described above. Significant effects for PCs that explained only a small percentage of shape variation (≤6.42%) can be found in Supplementary Figs S3–5.

### Gonopodium morphology and length

We assessed morphological information on gonopodium tip structures of *n* = 183 males from eight populations (12 to 35 individuals per population) as we missed to assess gonopodium morphology in the Xiamen and Nanjing populations. Because the male gonopodium is a delicate organ, we cut the entire gonopodium and photographed the distal tip laterally (left side) at 100× magnification using an Optec B 302 microscope equipped with an Optec TP510 CCD camera (both from Optec Instrument Co. ltd., Chongqing, China). We used 51 homologous landmarks described by Heinen-Kay and Langerhans^54^ to capture morphological variation (Fig. 1c). Using similar Procrustes analyses and PCA procedures as described above, we obtained nine PCs that cumulatively explained 89.93% of the total variance. We conducted MANCOVAs using morphology-related PCs as dependent variables, while including log_10_-transformed centroid size, total gonopodium length (determined during the assessment of body shape information, see above) and both climate-related PCs as covariates. We performed *post-hoc* ANCOVAs to determine the source(s) of variation in case of significant model terms. We subjected the data on gonopodium lengths (from all 10 populations) to an ANCOVA, using standard length and the two climatic PCs as covariates.

### Data availability

The datasets generated and/or analyzed for the current study are available from the corresponding author on reasonable request.

## Electronic supplementary material

Supplementary information
