# Supplementary material for: Natural and sexual selection drive multivariate phenotypic divergence along climatic gradients in an invasive fish
Source: Sci Rep. 2018 Jul 24;8:11164. doi: 10.1038/s41598-018-29254-4 (PMC6057953; doi:10.1038/s41598-018-29254-4)

Supplementary Information

Natural and sexual selection drive multivariate phenotypic divergence along climatic gradients in an invasive fish

Xu Ouyang, Jiancao Gao, Meifeng Xie, Binghua Liu, Linjun Zhou, Bojian Chen, Jonas Jourdan, Rüdiger Riesch, and Martin Plath

Table S1 **Standard population genetic parameters for the 10 invasive *G. affinis* populations included in this study.** For each population and locus, observed (H_O) and expected (H_E) heterozygosities as well as allelic richness (A) are presented. Zero values for estimates of heterozygosity indicate that the locus was monomorphic in this population. P-values < 0.05 indicate deviations from Hardy–Weinberg equilibrium. For site abbreviations see Table 3.

Population	Estimate	Gaaf7	Gaaf9	Gaaf10	Gaaf11	Gaaf13	Gaaf15	Gaaf16	Gaaf22	Gafu1	Gafu2	Gafu3	Gafu4	Gafu6	Gafu7	Mf-13	Mean across loci
AK	H_O	0.3	0.35	0.45	0.56	0.65	0.45	0.05	0.8	0.5	0.8	0.8	0.55	0.5	0.55	0.45	0.52
	H_E	0.55	0.31	0.64	0.62	0.55	0.38	0.05	0.68	0.58	0.73	0.77	0.86	0.57	0.55	0.51	0.56
	P	0.01	1	0.01	0.11	1	1	1	0.67	0.3	0.84	0.27	< 0.001	0.18	0.38	0.67	0.5
	A	7.93	2.95	4.61	3.72	4.53	2.88	1.65	3.96	2.96	5.3	5.3	10.8	2.96	2.99	2	4.3
BD	H_O	0.79	0.21	0.06	0.5	1	0.58	0.53	0.47	0.58	0.68	0.74	0.63	0.32	0.21	0.53	0.52
	H_E	0.79	0.19	0.54	0.54	0.84	0.59	0.51	0.59	0.55	0.56	0.7	0.79	0.34	0.59	0.44	0.57
	P	0.76	1	< 0.001	0.46	0.11	0.81	1	0.35	0.54	0.32	0.81	0.01	1	< 0.001	0.61	0.52
	A	5.66	1.99	5.97	3.72	8.13	3	2	3	2.99	4.37	4.87	7.99	2	5.97	2	4.24
BH	H_O	0.78	0.11	0.41	0.62	0.61	0.72	0.44	0.67	0.17	0.5	0.29	0.24	0.18	0.35	0.24	0.42
	H_E	0.7	0.29	0.58	0.68	0.54	0.61	0.51	0.64	0.53	0.4	0.52	0.32	0.34	0.75	0.21	0.51
	P	< 0.001	0.04	0.02	0.37	0.61	0.7	0.65	1	< 0.001	0.63	0.01	0.12	0.1	< 0.001	1	0.35
	A	3.72	2	4.85	4	3.72	3	2	3	5.71	2.72	6.69	3.84	2	8.65	2	3.86
CD	H_O	0.53	0.16	0.53	0.65	0.74	0.74	0.63	0.74	0.42	0.68	0.63	0.58	0.47	0.84	0.42	0.58
	H_E	0.71	0.37	0.71	0.7	0.74	0.56	0.53	0.73	0.56	0.74	0.72	0.83	0.67	0.72	0.4	0.65
	P	0.03	0.03	0.3	0.85	0.38	0.27	0.66	0.77	0.25	0.13	0.02	0.03	0.02	0.93	1	0.38
	A	3.91	4.68	3.97	3.95	7.22	2.91	3.59	3.99	2.91	4.68	7.17	10.2	3.97	3.99	2	4.61
HU	H_O	0.74	0.2	0.25	0.89	0.72	0.7	0.3	0.83	0.39	0.44	0.6	0.35	0.4	0.75	0.22	0.52
	H_E	0.81	0.53	0.56	0.74	0.69	0.51	0.52	0.61	0.52	0.51	0.86	0.71	0.67	0.67	0.2	0.61
	P	0.03	< 0.001	0.01	0.01	0.36	0.28	0.03	0.23	0.31	0.12	< 0.001	< 0.001	0.01	0.69	1	0.21

	A	6.96	6.51	6.28	4.72	6.29	3.53	2.65	3.71	2.72	5.5	9.66	8.7	7.14	3.88	2	5.35
XM	H_O	0.4	0.6	0.7	0.7	0.5	0.3	0.25	0.55	0.25	0.7	0.05	0.65	0.5	0.35	0.6	0.47
	H_E	0.57	0.65	0.67	0.51	0.58	0.38	0.45	0.71	0.22	0.64	0.54	0.62	0.69	0.3	0.49	0.54
	P	0.12	0.56	0.48	0.17	0.54	0.54	0.12	0.22	1	0.03	< 0.001	1	0.2	1	0.38	0.42
	A	3.64	3.65	3.99	2	3.95	2	2	3.99	2	3.88	6.52	3	3.96	2	2	3.24
CZ	H_O	0.6	0.45	0.6	0.2	0.75	0.3	0.4	0.6	0.35	0.8	0.25	0.6	0.75	0.55	0.6	0.52
	H_E	0.81	0.43	0.56	0.18	0.72	0.27	0.38	0.68	0.41	0.74	0.34	0.83	0.75	0.52	0.47	0.54
	P	0.01	1	0.1	1	0.85	1	1	0.37	0.59	0.99	0.15	0.01	0.51	1	0.33	0.59
	A	5.64	2.88	3.95	1.99	6.42	2.87	2	3	2	4	2.88	7.9	4.65	2.65	2	3.66
HA	H_O	0.3	0.45	0.45	0.65	0.65	0.35	0.8	0.65	0.4	0.7	0.7	0.55	0.55	0.6	0.05	0.52
	H_E	0.59	0.48	0.56	0.48	0.69	0.5	0.55	0.64	0.43	0.61	0.74	0.8	0.58	0.58	0.05	0.55
	P	< 0.001	0.86	0.07	0.16	0.08	0.2	0.04	0.62	1	0.33	0.18	< 0.001	0.06	0.31	1	0.33
	A	7.39	3.61	3.53	2	3.88	2	2.88	3.93	2	4.58	6.17	10.4	3.53	3.65	1.65	4.08
NJ	H_O	0.35	0.5	0.5	0.6	0.7	0.6	0.65	0.6	0.35	0.8	0.9	0.5	0.5	0.65	0.05	0.55
	H_E	0.77	0.44	0.5	0.51	0.67	0.51	0.58	0.66	0.48	0.68	0.79	0.83	0.57	0.58	0.05	0.58
	P	< 0.001	1	0.37	0.65	0.68	0.65	1	0.48	0.34	0.44	0.63	< 0.001	0.41	0.56	1	0.55
	A	9.3	2.65	5.3	2	4.3	2	3	3	2	4	6.54	10.76	4.18	3.93	1.65	4.31
LS	H_O	0.3	0.65	0.75	< 0.001	0.7	0.65	0.35	0.85	0.6	0.5	0.75	0.53	0.85	0.55	0.45	0.57
	H_E	0.28	0.5	0.69	< 0.001	0.69	0.51	0.45	0.73	0.57	0.53	0.76	0.68	0.69	0.66	0.36	0.54
	P	1	0.36	< 0.001	< 0.001	0.55	0.37	0.35	0.95	0.36	0.41	0.2	0.01	0.8	0.04	0.53	0.4
	A	4.07	2	4.3	1	6.03	2	2	4.65	3	3	7.59	5.05	4.76	3.65	2	3.67
	H_O	0.51	0.37	0.47	0.54	0.7	0.54	0.44	0.68	0.4	0.66	0.57	0.52	0.5	0.54	0.36	0.52
Mean across populations	H_E	0.66	0.42	0.6	0.5	0.67	0.48	0.45	0.67	0.49	0.62	0.67	0.73	0.59	0.59	0.32	0.56
	A	8.35	4.89	9.18	3.54	8.36	2.96	2.59	4.61	3.91	4.86	10.24	11.35	5.77	6.4	2	5.93

Table S2 Tests for genetic bottlenecks in the ten study populations under three microsatellite mutation models (see main text). L_E: expected number of loci with heterozygote excess; L_O: observed number of loci with heterozygote excess; P_W: probability value from two-tailed Wilcoxon signed-rank tests for $L_O > L_E$.

Population	IAM			TPM			SMM		
	L_E	L_O	P_W	L_E	L_O	P_W	L_E	L_O	P_W
AK	8.38	15	< 0.001	8.65	13	< 0.001	8.75	12	0.026
BD	8.60	13	< 0.001	8.73	11	0.008	8.77	10	0.639
BH	8.40	15	< 0.001	8.60	14	< 0.001	8.72	9	0.064
CD	8.67	15	< 0.001	8.84	13	< 0.001	8.87	11	0.073
CZ	8.44	13	< 0.001	8.74	13	0.018	8.90	11	0.121
HA	8.65	15	< 0.001	8.85	11	0.001	8.93	10	0.421
HU	8.84	15	< 0.001	8.90	13	0.008	8.93	7	1.000
LS	8.22	12	0.002	8.54	9	0.208	8.58	8	0.978
NJ	8.59	15	< 0.001	8.83	14	0.001	8.94	13	0.035
XM	8.51	15	< 0.001	8.88	15	< 0.001	9.07	10	0.010

Table S3 Results from (multivariate) analyses of covariance (M)ANCOVA and ANOVA. Depicted are the results of analyses of covariance similar to those shown in the main text (Table 2), but in this case no climatic information was included. Specifically, we included ‘population’ as a fixed factor and ‘standard length’ (SL), ‘centroid size’, and ‘stage of development’ as covariates where applicable. Statistically significant effects are shown in bold font. Relative variance explained was calculated from Wilk’s partial η^2.

	Source	d.f.	*F*	*P*	Partial η^2	Variance explained [%]
(*a*) Male standard length	**Population**	**9**	**56.665**	**< 0.001**	—	—
	Error	174	—	—	—	—
(*b*) Female standard length	**Population**	**9**	**41.425**	**< 0.001**	—	—
	Error	181	—	—	—	—
(*c*) Male life-history traits	**SL**	**3**	**330.067**	**< 0.001**	**0.853**	**100**
	**Population**	**27**	**20.778**	**< 0.001**	**0.519**	**60.84**
	Error	171	—	—	—	—
(*d*) Female life-history traits	**SL**	**6**	**140.189**	**< 0.001**	**0.829**	**100**
	**Population**	**54**	**12.725**	**< 0.001**	**0.385**	**46.44**
	Stage of development	6	2.010	0.068	0.065	7.84
	Error	174	—	—	—	—
(*e*) Male morphology-related PCs	**Centroid size**	**10**	**20.293**	**< 0.001**	**0.543**	**100**
	**Population**	**90**	**9.441**	**< 0.001**	**0.337**	**62.06**
	Error	171	—	—	—	—
(*f*) Female morphology-related PCs	**Population**	**90**	**11.771**	**< 0.001**	**0.361**	**100**
	**Centroid size**	**10**	**9.355**	**< 0.001**	**0.329**	**91.14**
	Error	191	—	—	—	—
(*g*) Gonopodium morphology-related PCs	**Centroid size**	**9**	**11.332**	**< 0.001**	**0.382**	**100**
	**Gonopodium length**	**9**	**8.652**	**< 0.001**	**0.321**	**84.03**
	**Population**	**63**	**4.808**	**< 0.001**	**0.202**	**52.88**
	Error	165	—	—	—	—
(*h*) Gonopodium length	**SL**	**1**	**328.209**	**< 0.001**	**0.637**	**100**
	**Population**	**9**	**7.724**	**< 0.001**	**0.271**	**42.54**
	Error	187	—	—	—	—

Table S4 Summary information of life-history parameters. Presented are means (±SD) of raw data collected from (*a*) $n = 184$ male and (*b*) $n = 191$ female *G. affinis* from 10 sampling sites across mainland China.

	Population	Sampling time	*N*	SL [mm]	Somatic lean weight [mg]	Fat content [%]	Fecundity	GSI [%]	RA [%]	Embryo lean weight [mg]	Embryo fat content [%]
(*a*) Male life-history traits	Baoding	Apr. 2015	23	20.18±1.13	26.92±4.47	2.84±1.55	—	1.91±0.52	—	—	—
	Ankang	Apr. 2016	29	19.90±2.07	27.24±9.61	6.22±0.69	—	1.80±0.33	—	—	—
	Nanjing	Jun. 2015	4	16.33±0.49	13.39±1.85	1.46±0.84	—	2.27±0.31	—	—	—
	Chengdu	Jun. 2016	11	16.22±1.01	14.06±3.07	2.68±1.05	—	2.54±0.75	—	—	—
	Huzhou	Apr. 2015	24	23.85±1.34	40.03±6.74	1.31±0.87	—	1.94±0.45	—	—	—
	Hangzhou	Apr. 2016	25	22.50±1.31	42.78±7.90	2.19±0.47	—	1.91±0.46	—	—	—
	Lishui	Sep. 2016	25	18.33±0.98	17.76±4.47	5.31±0.84	—	2.30±0.60	—	—	—
	Xiamen	Aug. 2015	11	16.54±0.74	15.09±3.01	1.85±0.58	—	1.61±0.45	—	—	—
	Chaozhou	Apr. 2016	13	18.81±1.53	18.72±5.02	4.00±0.71	—	2.93±1.03	—	—	—
	Beihai	Apr. 2015	19	20.14±1.32	25.59±5.06	2.42±0.85	—	2.60±0.48	—	—	—
(*b*) Female life-history traits	Baoding	Apr. 2015	14	24.60±1.82	63.53±7.74	3.12±1.03	6.64±2.59	—	5.29±2.73	0.75±0.35	6.39±3.92
	Ankang	Apr. 2016	23	28.20±3.51	90.71±46.71	6.82±1.52	39.39±20.46	—	15.69±6.52	0.60±0.20	3.17±1.68
	Nanjing	Jun. 2015	17	22.65±3.90	39.01±26.85	2.55±0.45	17.29±10.88	—	14.48±11.58	0.58±0.31	1.57±1.63
	Chengdu	Jun. 2016	9	26.34±6.13	72.15±54.36	1.36±0.54	40.89±21.35	—	23.74±5.97	0.53±0.13	1.24±0.96
	Huzhou	Apr. 2015	25	31.19±2.04	101.05±20.71	4.41±0.89	42.96±12.35	—	25.26±5.26	0.91±0.33	2.17±1.24
	Hangzhou	Apr. 2016	24	28.86±1.50	92.77±15.68	5.47±2.76	29.75±8.59	—	24.28±4.10	1.04±0.12	6.25±4.67
	Lishui	Sep. 2016	23	20.84±1.80	34.42±8.84	5.18±1.91	5.39±2.37	—	12.56±4.59	0.89±0.17	11.30±4.72
	Xiamen	Aug. 2015	7	19.74±1.73	26.53±6.04	2.93±1.18	4.43±2.70	—	8.74±6.38	0.64±0.32	3.35±2.43
	Chaozhou	Apr. 2016	24	27.75±1.67	63.28±16.10	4.46±0.83	25.75±9.32	—	20.91±7.44	0.87±0.22	3.27±1.57
	Beihai	Apr. 2015	25	26.90±1.61	63.90±13.04	3.60±1.52	14.68±5.67	—	20.30±3.99	1.16±0.16	4.08±2.36

Table S5 Results of *post-hoc* ANCOVAs. We conducted ANCOVAs on (*a*) male and (*b*) female life-history traits, (*c*) male and (*d*) female morphology-related PCs, and (*e*) gonopodium morphology-related PCs to identify the source(s) of variation in case of significant effects in our main MANCOVA models (Table 2). Relative variance explained was calculated using Wilk's partial η^2 only for models that showed significant model terms. Statistically significant effects are highlighted in bold font.

Model	Source	Dependent Variable	df	F	P	Partial η^2	Variance explained [%]
(*a*) Male life-history traits	SL	**Somatic lean weight**	**1**	**1751.1**	**< 0.001**	**0.907**	**100**
		**Fat content**	**1**	**17.236**	**< 0.001**	**0.088**	**9.7**
		GSI	1	1.106	0.294	0.006	0.66
	Climatic PC1	Somatic lean weight	1	0.678	0.411	0.004	0.44
		**Fat content**	**1**	**41.383**	**< 0.001**	**0.188**	**20.73**
		**GSI**	**1**	**18.615**	**< 0.001**	**0.094**	**10.36**
	Climatic PC2	Somatic lean weight	1	3.72	0.055	0.02	2.21
		**Fat content**	**1**	**39.542**	**< 0.001**	**0.181**	**19.96**
		GSI	1	1.003	0.318	0.006	0.66
	Climatic PC1 × PC2	Somatic lean weight	1	0.63	0.428	0.004	0.44
		**Fat content**	**1**	**74.95**	**< 0.001**	**0.295**	**32.52**
		GSI	1	2.297	0.131	0.013	1.43
		Error	179	—	—	—	—
(*b*) Female life-history traits	SL	**Somatic lean weight**	**1**	**1631.9**	**< 0.001**	**0.898**	**100**
		**Embryo lean weight**	**1**	**10.811**	**0.001**	**0.055**	**6.15**
		**Fat content**	**1**	**5.883**	**0.016**	**0.031**	**3.43**
		**Embryo fat content**	**1**	**6.925**	**0.009**	**0.036**	**4.02**
		**RA**	**1**	**35.42**	**< 0.001**	**0.161**	**17.89**
		**Fecundity**	**1**	**358.49**	**< 0.001**	**0.66**	**73.45**
	Stage of development	Somatic lean weight	1	1.668	0.198	0.009	0.99
		Embryo lean weight	1	0.543	0.462	0.003	0.33
		Fat content	1	0.405	0.525	0.002	0.24
		Embryo fat content	1	0.003	0.959	< 0.001	< 0.01

		RA	1	0.042	0.838	< 0.001	0.03
		Fecundity	1	2.642	0.106	0.014	1.57
	Climatic PC1	**Somatic lean weight**	**1**	**20.606**	**< 0.001**	**0.100**	**11.16**
		**Embryo lean weight**	**1**	**9.579**	**0.002**	**0.049**	**5.48**
		**Fat content**	**1**	**27.832**	**< 0.001**	**0.131**	**14.56**
		Embryo fat content	1	3.654	0.057	0.019	2.16
		**RA**	**1**	**28.754**	**< 0.001**	**0.135**	**14.98**
		Fecundity	1	0.402	0.527	0.002	0.24
	Climatic PC2	Somatic lean weight	1	1.101	0.295	0.006	0.66
		**Embryo lean weight**	**1**	**39.969**	**< 0.001**	**0.178**	**19.78**
		Fat content	1	0.375	0.541	0.002	0.23
		**Embryo fat content**	**1**	**8.797**	**0.003**	**0.045**	**5.06**
		RA	1	1.301	0.256	0.007	0.78
		**Fecundity**	**1**	**39.263**	**< 0.001**	**0.175**	**19.5**
	Climatic PC1 × PC2	Somatic lean weight	1	0.238	0.627	0.001	0.14
		**Embryo lean weight**	**1**	**4.664**	**0.032**	**0.025**	**2.74**
		**Fat content**	**1**	**44.401**	**< 0.001**	**0.194**	**21.55**
		Embryo fat content	1	0.067	0.797	< 0.001	0.04
		**RA**	**1**	**12.181**	**0.001**	**0.062**	**6.88**
		Fecundity	1	0.095	0.758	0.001	0.06
		Error	185	—	—	—	—
(*c*) Male morphology-related PCs	Centroid size	**PC1**	**1**	**12.928**	**< 0.001**	**0.065**	**32.51**
		PC2	1	2.084	0.151	0.011	5.42
		**PC3**	**1**	**47.616**	**< 0.001**	**0.204**	**100**
		PC4	1	0.671	0.414	0.004	1.97
		PC5	1	0.555	0.457	0.003	1.48
		PC6	1	0.636	0.426	0.003	1.48
		PC7	1	0.006	0.939	<0.001	—
		PC8	1	0.17	0.681	0.001	0.49
		PC9	1	1.876	0.172	0.01	4.93
		**PC10**	**1**	**7.936**	**0.005**	**0.041**	**20.2**
	Climatic PC1	**PC1**	**1**	**20.323**	**< 0.001**	**0.099**	**48.28**
		PC2	1	3.175	0.076	0.017	8.37
		**PC3**	**1**	**21.126**	**< 0.001**	**0.102**	**50.25**
		**PC4**	**1**	**8.175**	**0.005**	**0.042**	**20.69**

		PC5	1	0.029	0.866	<0.001	—
		PC6	1	1.456	0.229	0.008	3.94
		PC7	1	0.533	0.466	0.003	1.48
		PC8	1	3.499	0.063	0.018	8.87
		PC9	1	1.231	0.269	0.007	3.45
		**PC10**	**1**	**6.412**	**0.012**	**0.033**	**16.26**
	Climatic PC2	**PC1**	**1**	**40.529**	**<0.001**	**0.179**	**88.18**
		PC2	1	1.221	0.271	0.007	3.45
		PC3	1	1.712	0.192	0.009	4.43
		**PC4**	**1**	**7.047**	**0.009**	**0.037**	**17.73**
		PC5	1	0.473	0.493	0.003	1.48
		PC6	1	0.599	0.44	0.003	1.48
		PC7	1	0.686	0.409	0.004	1.97
		PC8	1	0.635	0.427	0.003	1.48
		**PC9**	**1**	**11.694**	**0.001**	**0.059**	**29.06**
		PC10	1	0.434	0.511	0.002	0.99
	Climatic PC1 × PC2	**PC1**	**1**	**47.214**	**< 0.001**	**0.202**	**99.51**
		**PC2**	**1**	**7.143**	**0.008**	**0.037**	**18.23**
		PC3	1	1.145	0.286	0.006	**2.96**
		**PC4**	**1**	**11.59**	**0.001**	**0.059**	**29.06**
		PC5	1	1.489	0.224	0.008	**3.94**
		PC6	1	3.019	0.084	0.016	7.88
		PC7	1	0.423	0.516	0.002	0.99
		PC8	1	0.195	0.659	0.001	0.49
		PC9	1	0.094	0.759	< 0.001	—
		PC10	1	2.075	0.151	0.011	5.42
		Error	186	—	—	—	—
(*d*) Female morphology-related PCs	Centroid size	PC1	1	0.004	0.947	< 0.001	—
		PC2	1	1.127	0.29	0.005	2.15
		PC3	1	0.731	0.394	0.004	1.72
		**PC4**	**1**	**5.777**	**0.017**	**0.027**	**11.59**
		PC5	1	0.101	0.751	< 0.001	—
		PC6	1	1.068	0.303	0.005	2.15
		**PC7**	**1**	**5.747**	**0.017**	**0.027**	**11.59**
		PC8	1	1.789	0.183	0.009	3.86
		PC9	**1**	0.153	0.696	0.001	0.43
		**PC10**	**1**	**7.197**	**0.008**	**0.034**	**14.59**
	Climatic PC1	**PC1**	**1**	**5.886**	**0.016**	**0.028**	**12.02**
		**PC2**	**1**	**38.425**	**< 0.001**	**0.157**	**67.38**
		PC3	1	1.524	0.218	0.007	3
		**PC4**	**1**	**10.758**	**0.001**	**0.05**	**21.46**
		**PC5**	**1**	**6.706**	**0.01**	**0.032**	**13.73**
		PC6	1	2.814	0.095	0.013	5.58

		PC7	1	0.019	0.891	< 0.001	—
		PC8	1	0.32	0.572	0.002	0.86
		PC9	1	0.392	0.532	0.002	0.86
		**PC10**	**1**	**10.163**	**0.002**	**0.047**	**20.17**
	Climatic PC2	**PC1**	**1**	**59.354**	**< 0.001**	**0.224**	**96.14**
		**PC2**	**1**	**21.169**	**< 0.001**	**0.093**	**39.91**
		PC3	1	2.002	0.159	0.010	4.29
		**PC4**	**1**	**13.787**	**< 0.001**	**0.063**	**27.04**
		PC5	1	0.698	0.404	0.003	1.29
		PC6	1	0.276	0.6	0.001	0.43
		PC7	1	0.319	0.573	0.002	0.86
		PC8	1	0.989	0.321	0.005	2.15
		PC9	1	0.357	0.551	0.002	0.86
		PC10	1	0.033	0.856	< 0.001	—
	Climatic PC1 × PC2	PC1	1	3.255	0.073	0.016	6.87
		**PC2**	**1**	**62.676**	**< 0.001**	**0.233**	**100**
		**PC3**	**1**	**7.199**	**0.008**	**0.034**	**14.59**
		PC4	1	1.152	0.284	0.006	2.58
		PC5	1	0.648	0.422	0.003	1.29
		PC6	1	1.05	0.307	0.005	2.15
		**PC7**	**1**	**4.625**	**0.033**	**0.022**	**9.44**
		PC8	1	0.132	0.717	0.001	0.43
		PC9	1	0.296	0.587	0.001	0.43
		PC10	1	3.471	0.064	0.017	7.3
		Error	191	—	—	—	—
(*e*) Gonopodium morphology-related PCs	Centroid size	**PC1**	**1**	**24.9**	**< 0.001**	**0.123**	**99.19**
		**PC2**	**1**	**18.52**	**< 0.001**	**0.095**	**76.61**
		**PC3**	**1**	**9.5**	**0.002**	**0.051**	**41.13**
		PC4	1	0.93	0.336	0.005	4.03
		PC5	1	0.01	0.924	< 0.001	—
		**PC6**	**1**	**15.08**	**< 0.001**	**0.079**	**63.71**
		**PC7**	**1**	**7.93**	**0.005**	**0.043**	**34.68**
		**PC8**	**1**	**22.16**	**< 0.001**	**0.111**	**89.52**
		PC9	1	0.1	0.751	0.001	0.81
	Gonopodium length	**PC1**	**1**	**25.14**	**< 0.001**	**0.124**	**100**
		PC2	1	0.12	0.733	0.001	0.81
		PC3	1	0.48	0.487	0.003	2.42
		PC4	1	0.17	0.678	0.001	0.81
		PC5	1	1.08	0.299	0.006	4.84
		**PC6**	**1**	**5.43**	**0.021**	**0.03**	**24.19**
		**PC7**	**1**	**6.62**	**0.011**	**0.036**	**29.03**
		**PC8**	**1**	**18.77**	**< 0.001**	**0.096**	**77.42**
		PC9	1	2.63	0.107	0.015	12.1

	Climatic PC1	**PC1**	**1**	**4.88**	**0.028**	**0.027**	**21.77**
		PC2	1	0.19	0.665	0.001	0.81
		PC3	1	1.83	0.177	0.01	8.06
		PC4	1	0.39	0.532	0.002	1.61
		**PC5**	**1**	**10.16**	**0.002**	**0.054**	**43.55**
		PC6	1	0.46	0.5	0.003	2.42
		PC7	1	1.92	0.168	0.011	8.87
		PC8	1	0.13	0.715	0.001	0.81
		**PC9**	**1**	**4.23**	**0.041**	**0.023**	**18.55**
	Climatic PC2	PC1	1	2.14	0.145	0.012	9.68
		PC2	1	1.98	0.161	0.011	8.87
		**PC3**	**1**	**4.99**	**0.027**	**0.027**	**21.77**
		PC4	1	0.48	0.489	0.003	2.42
		PC5	1	0.3	0.582	0.002	1.61
		PC6	1	3.6	0.059	0.02	16.13
		PC7	1	0.44	0.506	0.003	2.42
		**PC8**	**1**	**8.14**	**0.005**	**0.044**	**35.48**
		**PC9**	**1**	**4.11**	**0.044**	**0.023**	**18.55**
	Climatic PC1 × PC2	PC1	1	2.49	0.116	0.014	11.29
		PC2	1	0.57	0.45	0.003	2.42
		**PC3**	**1**	**4.84**	**0.029**	**0.027**	**21.77**
		PC4	1	2.79	0.097	0.015	12.1
		PC5	1	0.24	0.625	0.001	0.81
		PC6	1	1.88	0.173	0.010	8.06
		PC7	1	1.1	0.296	0.006	4.84
		PC8	1	0.01	0.944	< 0.001	—
		**PC9**	**1**	**5.95**	**0.016**	**0.033**	**26.61**
		Error	177	—	—	—	—

7

Table S6 Information on primers (forward: above, reverse: below) used to amplify 15 nuclear microsatellite loci. Ranges of allele sizes are given for the entire data set (i.e. across populations).

Group	Locus	Primer 5' - 3'	Repeat unit	Size range [bp]	Dye group	References
Mix 1	Gaaf11	ACTCAAGGCTGCCATACTGC<br>GGACTTAAGAGTGCCATACTGC	ACAG	134-178	HEX	75
	Gaaf13	ACTTGGTGGCAGATTTCAGG<br>AAGGAAACAACATGCTGGC	GATT	123-207	FAM	75
	Gaaf16	GTAGGTCATCTTCAATCTGGG<br>CTCCAGAGGCAGAATGTGTG	ATCC	223-283	HEX	75
	Gafu1	TCTGTTAGTGCTCAGCTGCAA<br>ATCAACAGCAGCCTCCTTCT	GA	104-108	FAM	73
	Gafu3	CTCAGCCGTCATTTAGTCTCAT<br>GCACATAACATGGAAACAGTAAAC	GT	234-280	ROX	73
Mix 2	Gaaf7	TCCATCCCATTATGACCACAG<br>GCACTTAGAAATGCCTCGC	AATC	139-235	ROX	75
	Gaaf9	GGTGCAAATCCGCAGCTTG<br>GGGAAATACTCCTGGACTCG	ACAG	222-262	HEX	75
	Gaaf15	TGCATGTGTGTTTGGTAAGG<br>GATCCCTGTTACACTGCTGG	AATG	137-161	HEX	75
	Gaaf22	ATGCGACCTGAAACTTCTGC<br>CCGAGGTCCTTGAGGTTTATAG	ATC	235-271	FAM	75
	Gafu2	CTCCAAACACACGTCCAATAATC<br>AGTTTCCCCAGCCGTTCAT	CA	143-171	FAM	73
	Gafu6	ACGAAGAGAGCAGCGGAGTTTTGG<br>CACCGGACAGACCAGCCTCA	GA	191-213	HEX	73
Mix 3	Gaaf10	GAACTGAACCACCCAAAGGC<br>TCCATCTGGAGACAGGTGTG	ATCC	236-320	HEX	75
	Gafu4	ACAACGGAGACCTGCTGGAGTGG<br>CGCGAACCGTCCGTTATCCGTA	CT	214-276	TAM RA	73
	Gafu7	CACAGAACAACACAGAAACTGGAGG<br>TGCCGATGGATGTTCCTGTTAG	AG	161-181	HEX	73
	Mf-13	AAAGGCTGCAAACAGTAAAAGTTA<br>GTCACAAATATAAGCCACAGAC	GT	154-156	FAM	74

Figure S1 **Additional information on male life-history divergence ('alternative' depiction of the interaction of both climate-related PCs; see main text).** As climate-related PC2 increases, male *G. affinis* from southern populations decreased fat content, while the trend was reversed in northern populations.

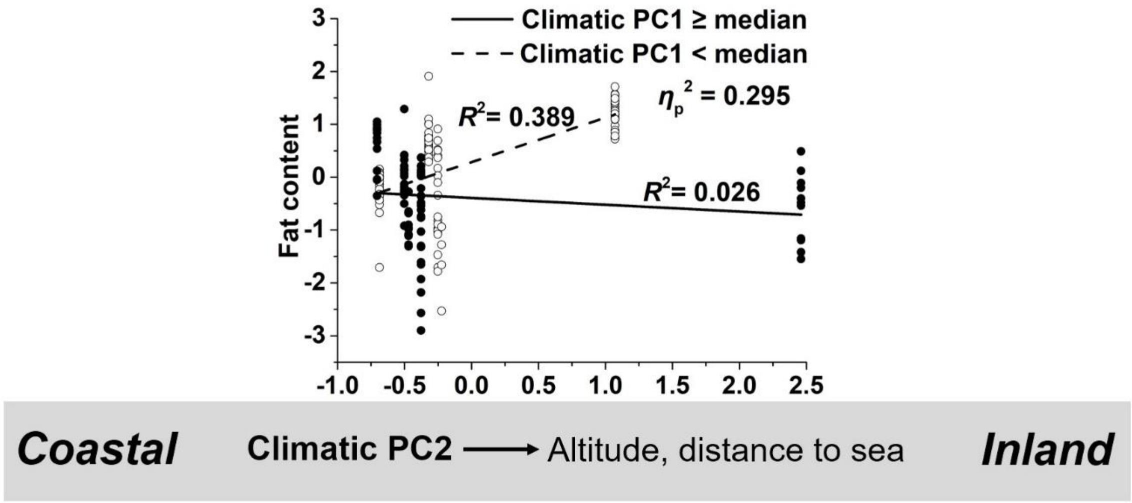

Figure S2 **Additional information on female life-history divergence ('alternative' depiction of the interaction of both climate-related PCs; see main text).** Southern populations decreased (*a*) fat content and increased (*b*) reproductive allocation (RA) and (*c*) embryo lean weight while the trend was reversed in northern populations with increasing values of climate-related PC2.

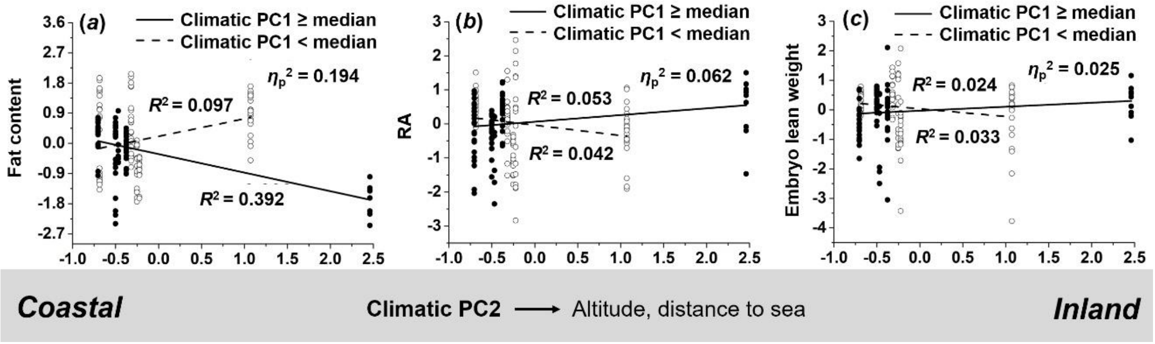

Figure S3 Additional information on variation of male morphology (PCs) along climatic PCs (statistically weak main effects and 'alternative' depictions of interaction terms; see main text). Increasing values of climatic PC1 resulted in (*a*) bigger heads and lower pectoral fins. Increasing values of climatic PC2 led to (*b*) more slender bodies, longer caudal peduncles and more anteriorly positioned gonopodia in southern populations but a reversed pattern in northern populations, (*c*) more upward-oriented pectoral and caudal fins in southern populations while the trend was reversed in northern populations, (*d*) more slender bodies, smaller heads, and decreased caudal peduncle lengths in southern populations but deeper bodies, bigger heads and increased peduncle lengths in northern populations, (*e*) increased caudal peduncle length and slender bodies.

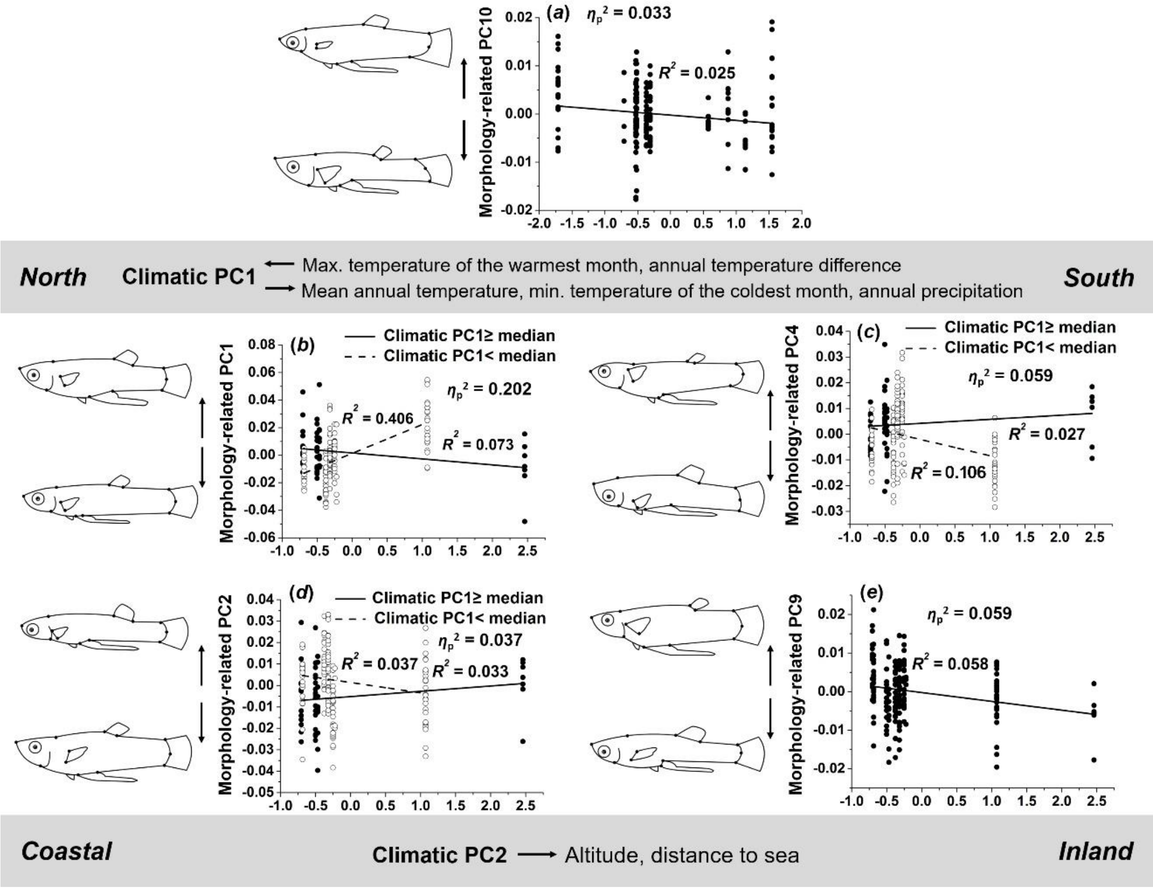

Figure S4 **Additional information on variation of female morphology (PCs) along climatic PCs (statistically weak main effects and 'alternative' depictions of interaction terms; see main text).** Increasing values of climatic PC2 led to (*a*) more slender bodies, smaller heads, more upward-oriented pectoral fins and caudal peduncles in southern populations while a reversed pattern emerged in northern populations, and (*b*) more slender bodies, bigger heads, downward-oriented pectoral fins, and shorter and more downward-oriented caudal peduncles in southern populations while the trend was reversed in northern populations, (*c*) smaller heads, longer caudal peduncles and decreased body depths in females from southern populations, with a tendency towards a reversed trend in northern populations. (*d*) As climatic PC1 increases, inland populations showed smaller heads, more slender bodies while the trend in coastal populations was reversed. Females showed (*e*) bigger heads, decreased caudal peduncle areas and more slender bodies with the increase of climatic PC1.

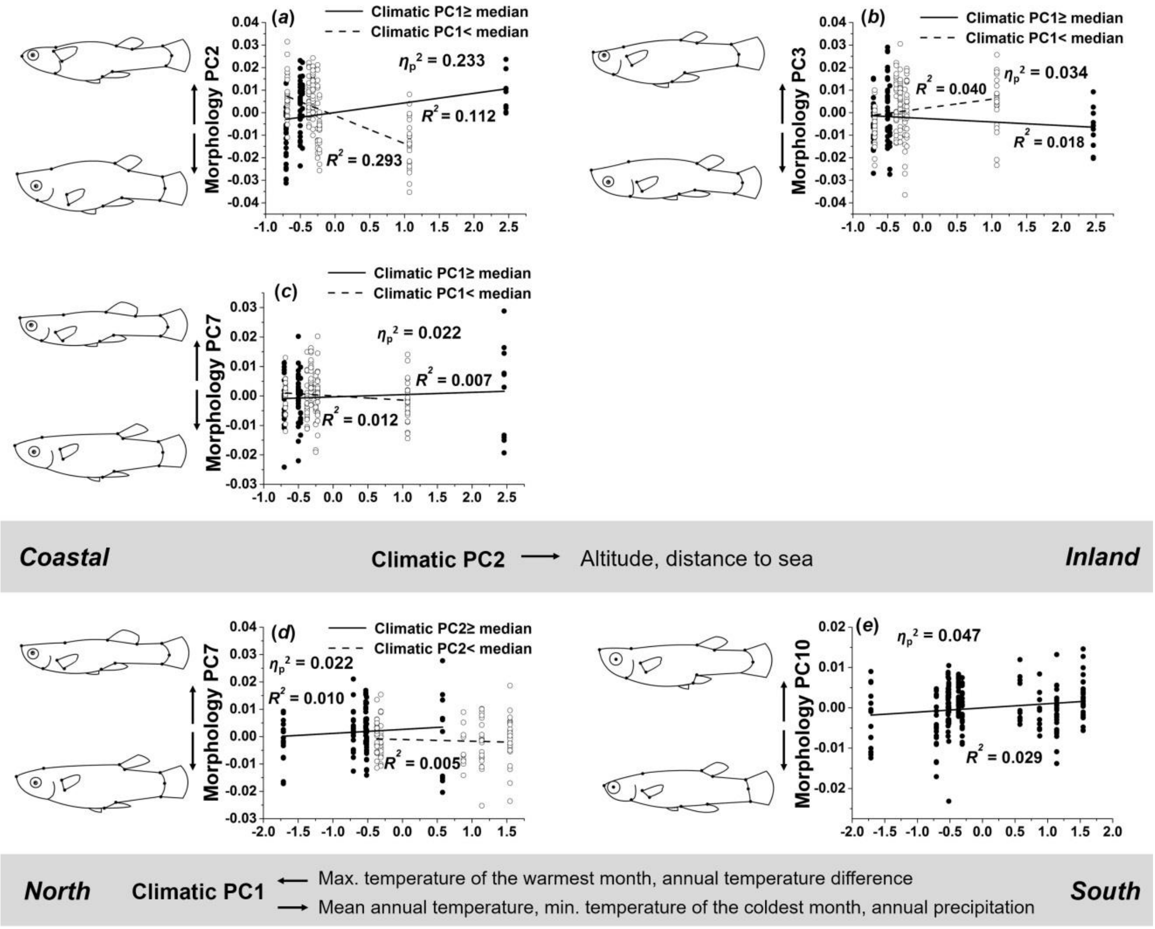

Figure S5 **Additional information on divergence of the male intromittant organ, the gonopodium (statistically weak main effects and 'alternative' depictions of interaction terms; see main text).** With increasing values of climatic PC2, male gonopodia had (*a*) smaller hooks and a narrower cavity between fin rays 4a and 4p, and a slightly increase trend showing (*b*) bigger hooks and a more slender overall shape. As climatic PC1 increases, male gonopodia showed (*c*) a slight bigger hooks and a more slender overall shape. The interaction effects on gonopodium PC9 showed (*d*) inland populations had bigger hooks and more slender gonopodia towards south while coastal populations had a very weak decreased pattern. With increasing values of climatic PC2, (*e*) southern populations possessed slender gonopodia with bigger hooks, while the trend was reversed in northern sites. (*f*) Males had a slight trend showing deeper gonopodia and looser spines, along with longer gonopodium tips from coast to inland, while the trend differed slightly between northern and southern populations.

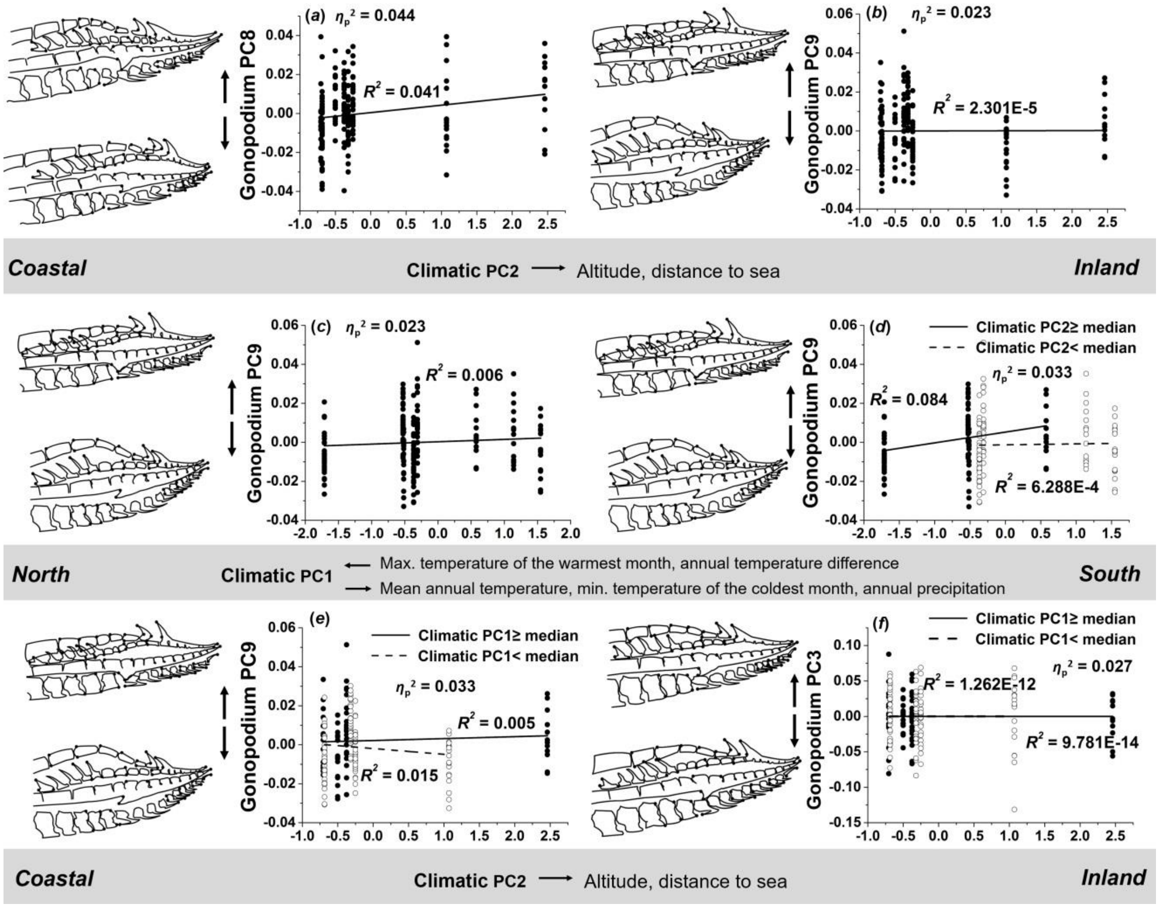

Supplement: Supplementary file 1 — Supplementary information [file 41598_2018_29254_MOESM1_ESM.pdf]
